# Integrated analysis of EGFR mutated non‐small cell lung cancer reveals two distinct molecular subtypes

**DOI:** 10.1002/ctm2.1431

**Published:** 2023-10-13

**Authors:** Menglin Bai, Leilei Wu, Mengyu Zhao, Peng Jin, Jingru Liu, Weiqing Wang, Xuetian Gao, Yanan Wang, Wei Chong, Jinming Yu, Hao Chen, Xue Meng

**Affiliations:** ^1^ Shandong University Cancer Center Jinan China; ^2^ Department of Radiation Oncology and Shandong Provincial Key Laboratory of Radiation Oncology Shandong Cancer Hospital and Institute Shandong First Medical University and Shandong Academy of Medical Sciences Jinan China; ^3^ Research Unit of Radiation Oncology Chinese Academy of Medical Sciences Jinan China; ^4^ Department of Radiation Oncology Shanghai Pulmonary Hospital Tongji University School of Medicine Shanghai China; ^5^ Cheeloo College of Medicine Shandong University Jinan China; ^6^ National Fire and Rescue Administration Tianjin Training Brigade Tianjin China; ^7^ Department of Gastrointestinal Surgery Key Laboratory of Engineering of Shandong Province Medical Science and Technology Innovation Center Shandong Provincial Hospital Affiliated to Shandong First Medical University Shandong First Medical University and Shandong Academy of Medical Sciences Jinan China; ^8^ Clinical Research Center of Shandong University Clinical Epidemiology Unit Qilu Hospital of Shandong University Jinan China

Dear Editor,

Epidermal growth factor receptor (EGFR) mutations predominantly drive non‐small cell lung cancer (NSCLC), particularly in individuals of East Asian descent.^1^, ^2^ Although EGFR‐tyrosine kinase inhibitors (TKIs), like Osimertinib, have enhanced survival for patients with EGFR‐mutated NSCLC,^3^ responses to EGFR‐TKIs vary, underlining heterogeneity within these tumours.^4^, ^5^, ^6^ Current genomic and transcriptomic understanding of EGFR‐mutant NSCLC is extensive, yet proteomic‐level analysis is lacking. Our study aims to unravel this heterogeneity by integrating transcriptomics, proteomics, and phosphoproteomics data of EGFR‐mutated NSCLC, revealing new biological subtypes and potential therapeutic avenues (Figure S1).

We utilized multi‐omics data from the Chen cohort^7^ (Table S1), comprising transcriptomics, proteomics, and phosphoproteomics, to establish molecular subtyping of EGFR‐mutant NSCLC. Two robust subtypes, S1 and S2, were identified, with proteomic and phosphoproteomic data significantly contributing to the clustering (Figure 1A,B, Figure S2A–E). Clinicopathological and genomic characteristics were similar across subtypes, but S2 tumours exhibited a higher incidence of co‐occurring TP53 mutations (Figure 1C). Using the random forest algorithm, we discerned distinct molecular signatures between the subtypes. (Figure 1D, Figure S3A).

**FIGURE 1:**
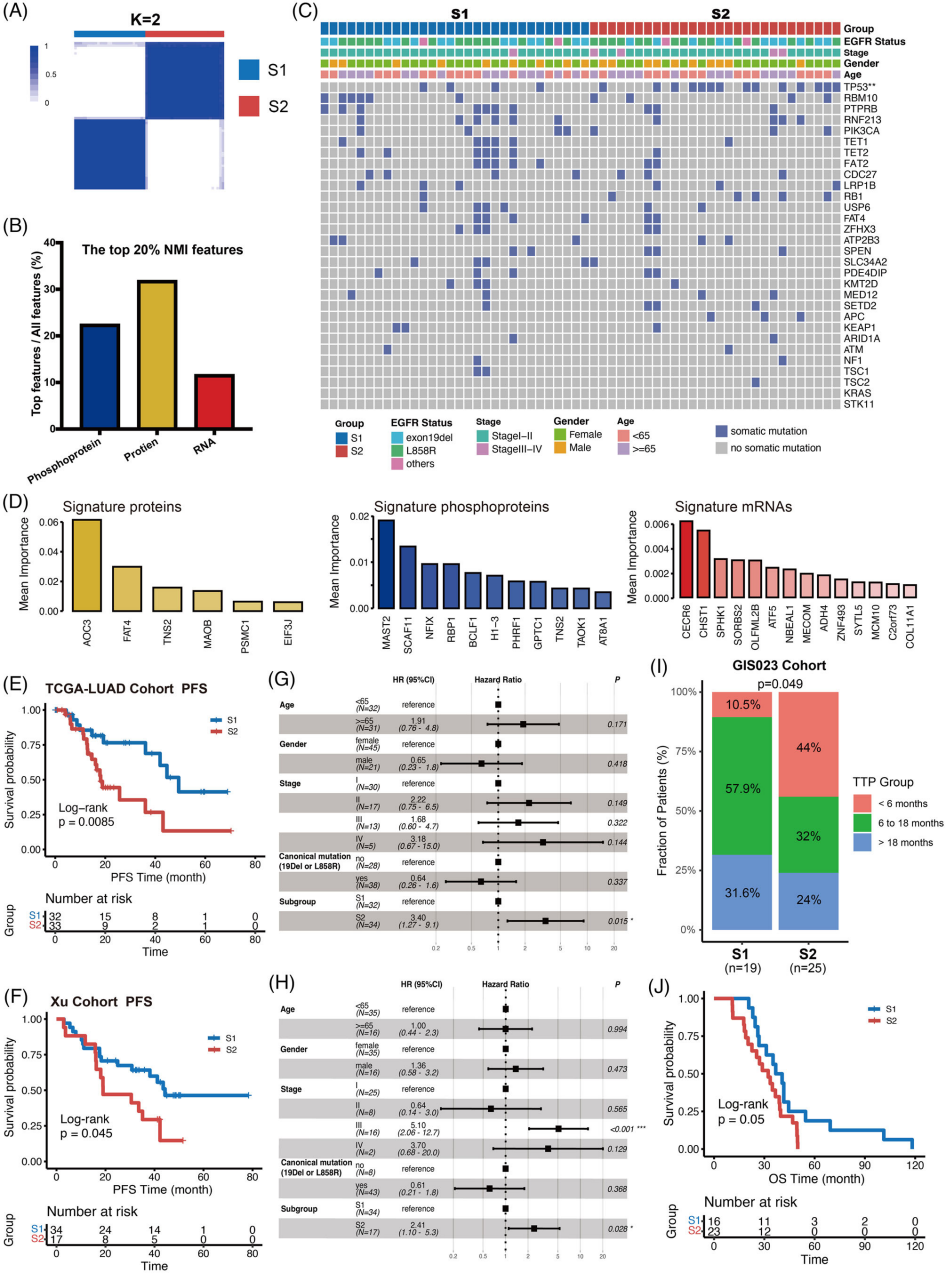
Integrative analysis of multi‐omics data of epidermal growth factor receptor (EGFR)‐mutant non‐small cell lung cancer (NSCLC) tumours reveals two distinct subtypes. (A) Consensus matrix of unsupervised clustering identified the best cluster number with *k* = 2. (B) Contribution of each data type to fused similarity network. (C) Clinical and genomic information of EGFR‐mutant patients in Chen cohort. *p < .05, **p < .01 and ***p < .001. (D) The most discriminative signatures of each layer of omic data (protein, phosphoprotein and mRNA respectively) selected by random forest algorithm. (E) Progression‐free survival (PFS) of the two EGFR‐mutant subtypes in TCGA cohort using Kaplan–Meier analysis. (F) PFS of the two EGFR‐mutant subtypes in Xu cohort using Kaplan–Meier analysis. (G) The association between the two EGFR‐mutant subtypes and PFS in TCGA cohort after being adjusted for age, gender, stage, mutation type using multivariate Cox model. (H) The association between the two EGFR‐mutant subtypes and PFS in Xu cohort after being adjusted for age, gender, stage, mutation type using multivariate Cox model. (I) Comparison of the proportion of time‐to‐progression (TTP) group in different EGFR‐mutant subtypes in GIS023 cohort (*p* = .049, chi‐square test). (J) Overall survival (OS) of the two EGFR‐mutant subtypes in GIS023 cohort using Kaplan‐Meier analysis. *p*‐Values in (E and F) were tested by log‐rank test.

To investigate the clinical relevance of our subtype classification, we then employed these signatures to predict EGFR‐mutant tumour subtyping in The Cancer Genome Atlas‐Lung Adenocarcinoma (TCGA‐LUAD) (*n* = 66), Xu (*n* = 51), and CPTAC (*n* = 40) cohorts^8^, ^9^ (Figure S3B–G, Table S2–S4). Initial prognostic analysis associated the S2 subtype with shorter progression‐free survival (PFS) and overall survival (OS) in both TCGA‐LUAD and Xu cohorts (Figure 1E, F, Figure S3H–J). Multivariate Cox regression analysis provided additional insights into the potential association of our clustering model with patient outcomes, independent of clinicopathological factors (Figure 1G, H). Analysis of disease‐specific survival (DSS), disease‐free interval (DFI) in TCGA‐LUAD cohort showed similar results (Figure S3K, L). Moreover, significant therapeutic benefits of EGFR‐TKIs regarding time to progression (TTP) and OS in the S1 subtype was observed in GIS‐023 cohort^10^ (Figure 1I‐J), highlighting the potential use of our subtype classification in patient stratification and precision diagnosis of EGFR‐mutant NSCLC. The absence of prognostic differences in EGFR‐wildtype NSCLC patients (TCGA‐LUAD *n* = 403; Xu *n* = 52), when classified by subtype‐specific signatures, further substantiated our classification's specificity to EGFR‐mutant NSCLC (Figure S3J‐L).

Next, we examined the somatic mutation landscape of two subtypes in TCGA‐LUAD cohorts, revealing a higher incidence of co‐occurring TP53 mutations in S2 tumours, corroborated by the CPTAC cohort (Figure 2A, Figure S4A). Relative to S1, the S2 subtype demonstrated increased C > G and C > T transitions and decreased C > A and T > A transitions, alongside a lower tumour mutation burden (TMB) (Figure 2B, C). Mutational signature analysis indicated S2 had increased mutations associated with APOBEC and dMMR, as well as elevated Cell‐cycle and Immune signature scores (Figure 2D, S4B‐E). S1 exhibited higher Arm SCNA levels (Figure 2E). The notable focal region amplifications in both subtypes were showed in Figure 2F.

**FIGURE 2:**
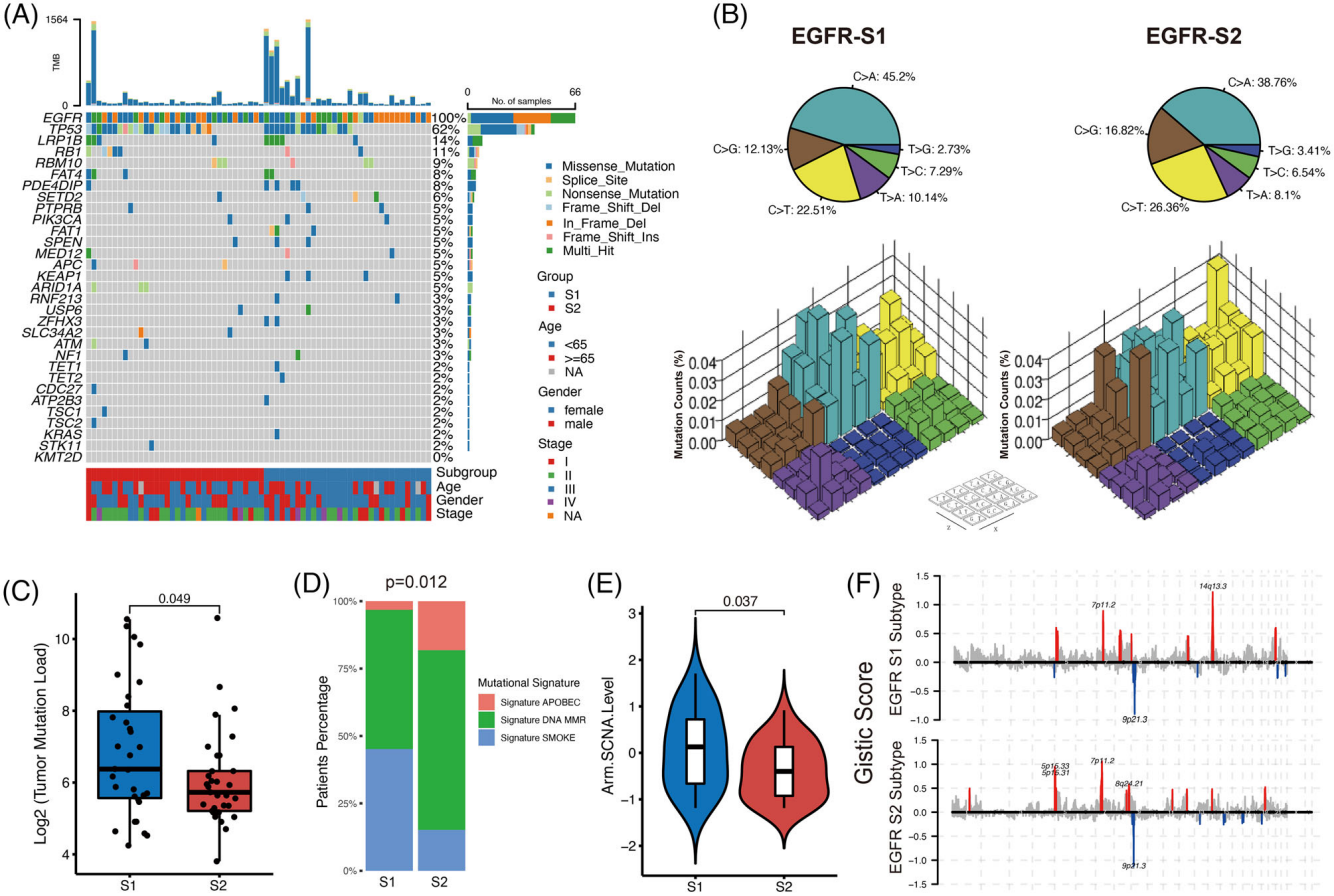
Tumour genomic variations in two epidermal growth factor receptor (EGFR)‐mutant non‐small cell lung cancer (NSCLC) subtypes. (A) Mutational landscape of EGFR mutant NSCLC tumours in TCGA cohort stratified by S1 and S2 subtypes. (B) (Upper) Pie chart showed the proportion of six major categories of nucleotide variations; (lower) Lego plot illustrates the 96 different nucleotide mutation patterns found in tumour samples. Single‐nucleotide substitutions were classified into six categories based on the 16 surrounding nucleotide bases. (C) Comparison of tumour mutation load in different EGFR‐mutant subtypes (*p* = .049, Wilcoxon test). (D) Proportion of extracted mutational signatures (APOBEC, SMOKE and DNA MMR‐related signatures) in different EGFR‐mutant subtypes (*p* = .012, chi‐square test). (E) Comparison of Arm level SCNA in different EGFR‐mutant subtypes (*p* = .037, Wilcoxon test). (F) Significant somatic copy number alterations including amplifications (red) or deletions (blue) in different EGFR‐mutant subtypes. Cytobands with GISTIC *q*‐value < .1 were labelled.

Furthering our understanding of the differential clinical outcomes between subtypes, we conducted pathway enrichment analyses on transcriptomic, proteomic, and phosphoproteomic levels. This analysis, based on a larger meta‐cohort (*n* = 99) created by merging the transcriptomic data of Chen and Xu cohorts, indicated that the S1 subtype was enriched in the P53 pathway, whereas the S2 subtype showed enrichment of MTORC1 signalling, G2M checkpoint, glycolysis, and MYC targets (Figure 3A). GSEA analysis confirmed these findings in both RNA and protein levels across Chen and TCGA‐LUAD cohorts (Figure 3B, S5A). Post‐translational modification (PTM) analysis associated the S1 subtype with the activation of kinase PKCZ, PKACA, and the S2 subtype with the activation of kinase CDK2, mammalian Target Of Rapamycin (mTOR), and CK2A1 (Figure 3C, Table S5). These results were further corroborated in the Kinase‐substrate enrichment analysis (KSEA) (Figure S5B, Table S6). Notably, the S2 subtype showed a significant up‐regulation of phosphosites associated with apoptosis inhibition, transcription induction, cell growth induction and cell cycle regulation (Figure 3D). Further analysis, using the DepMap dataset's large‐scale CRISPR screening, revealed 14 genes displaying notable dependency score differences between the subtypes (Figure S5D, E).

**FIGURE 3:**
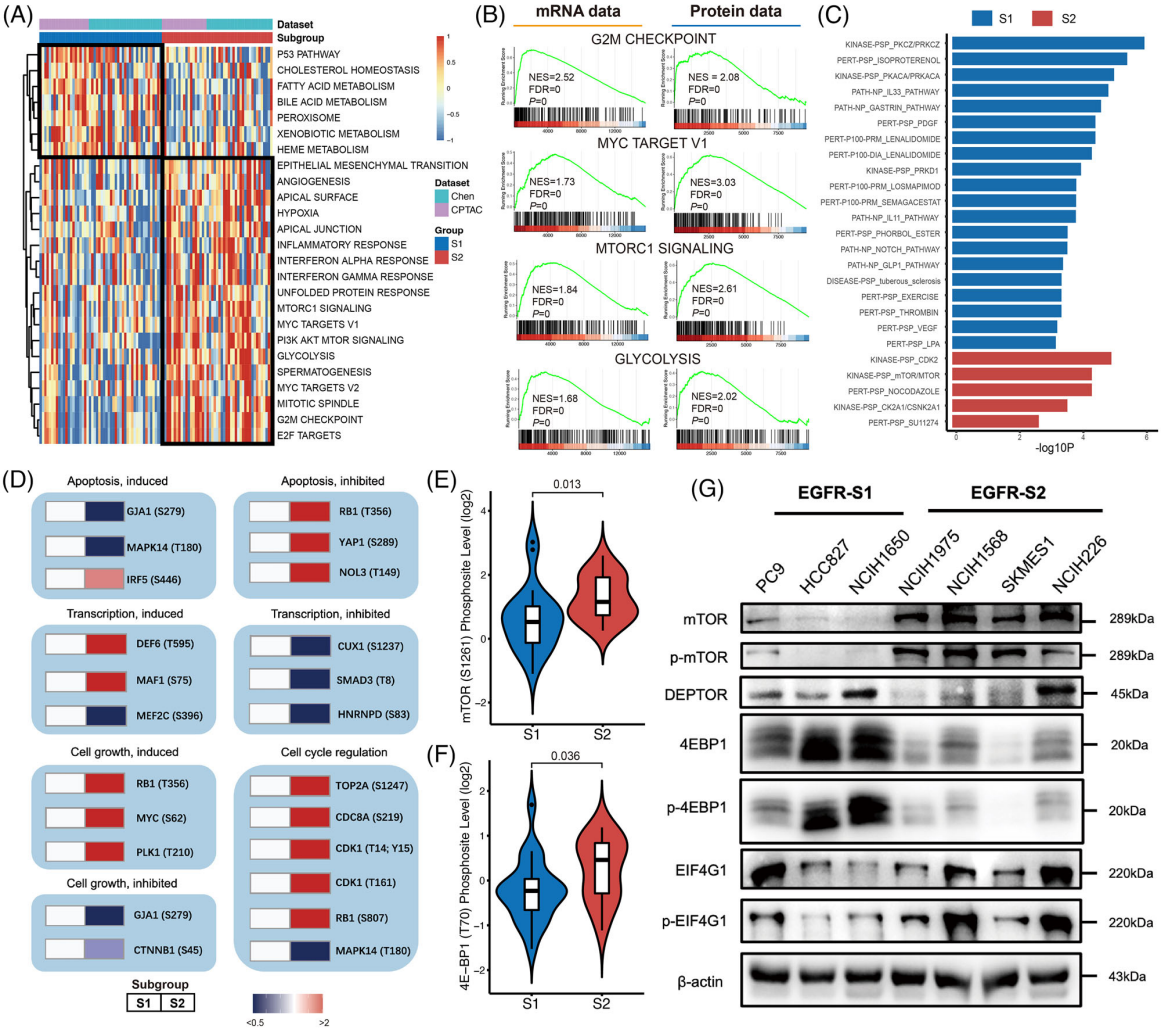
Pathways analysis in epidermal growth factor receptor (EGFR) mutant subtypes. (A) Heatmap shows the representative biological process in different EGFR‐mutant subtypes revealed by ssGSEA analysis at RNA level in the Chen‐CPTAC meta‐cohort. (B) GSEA enrichment plots of representative pathways in S2 subtype in Chen cohort. (C) Representative biological pathways of different subtypes revealed by PTM‐SEA analysis at phosphoprotein level in Chen cohort. (D) Annotation of differential phosphosites in two subtypes, retrieved from PhosphoSitePlus database. The color represents S2 verses S1. (E and F) Comparison of phospho‐mTOR (S1261) and phospho‐4E‐BP1 (T70) in different subtypes of EGFR mutant non‐small cell lung cancer (NSCLC) tumours in CPTAC cohort (*p* = .013 and .036 respectively, Wilcoxon test). (G) Western‐blot analysis of mTOR signalling pathway‐related proteins in selected EGFR‐mutant NSCLC cell lines from different subtypes.

Interestingly, we found S2 subtype showed consistent upregulation of the mTOR signalling pathway across transcriptomic, proteomic, and phosphoproteomic analyses. This was further validated in the CPTAC cohort, with notable upregulation of mTOR (S1261) and 4EBP1 (T70, inhibitory site) in S2 tumours (Figure 3E, F). Western blot analysis of EGFR‐mutant NSCLC cell lines mirrored this, with upregulated mTOR, phospho‐mTOR, mTOR‐related transcriptional factor EIF4G1, and phospho‐EIF4G1 in S2, and mTOR‐related inhibitory proteins, including DEPTOR, 4EBP1, and phospho‐4EBP1 upregulated in S1 (Figure 3G).

By examining the tumour immune microenvironment (TIME) between subtypes, we found immune‐related pathways and M1 macrophages enriched in S2 (Figure S5A–D). Immune subtype analysis indicated a predominance of Interferon‐gamma (IFN‐γ) Dominant and Wound Healing in S2 (Figure S5E). The tumour immune dysfunction and exclusion (TIDE) assessment revealed a heightened immunotherapy response potential in S2, given its pro‐inflammatory TIME (Figure S5F–I). Taken together, the differences in mutational landscape, biological pathways and TIME further supported the existence of intra‐tumour heterogeneity within EGFR‐mutant NSCLC.

Lastly, we aimed to discern subtype‐specific therapeutic agents for S2 subtype tumours by selecting proteins with significant expression differences between the subtypes and those significantly correlating with patient survival in the Xu‐cohort. A total of 52 up‐regulated and 41 down‐regulated proteins were identified in the S2 subtype. Utilizing these proteins as query signatures, we matched them to the connectivity map database, yielding six drugs with the highest negative connectivity scores: Palbociclib (CDK4/6 inhibitor), AKT‐inhibitor‐1‐2 (AKT inhibitor), BMS‐536924 (IGF‐1 inhibitor), PI‐103 (PI3K/mTOR inhibitor), AZD‐8055 (mTOR inhibitor) and Dactolisib (mTOR inhibitor) (Figure 4A, B, Table S7). In vitro validation using cell viability assay confirmed the efficacy of these drugs in EGFR‐mutant NSCLC cell lines, particularly inhibiting S2 proliferation (Figure 4C, S7C). Further validation via colony formation assays indicated a significant reduction in cell survival in the nanomolar or low micromolar range for the S2 subtype, whereas S1 subtype cell lines exhibited decreased sensitivity (Figure 4D, E). Still, observed individual variances in drug responses suggest the proposed pathways and drugs warrant further in vivo validation.

**FIGURE 4:**
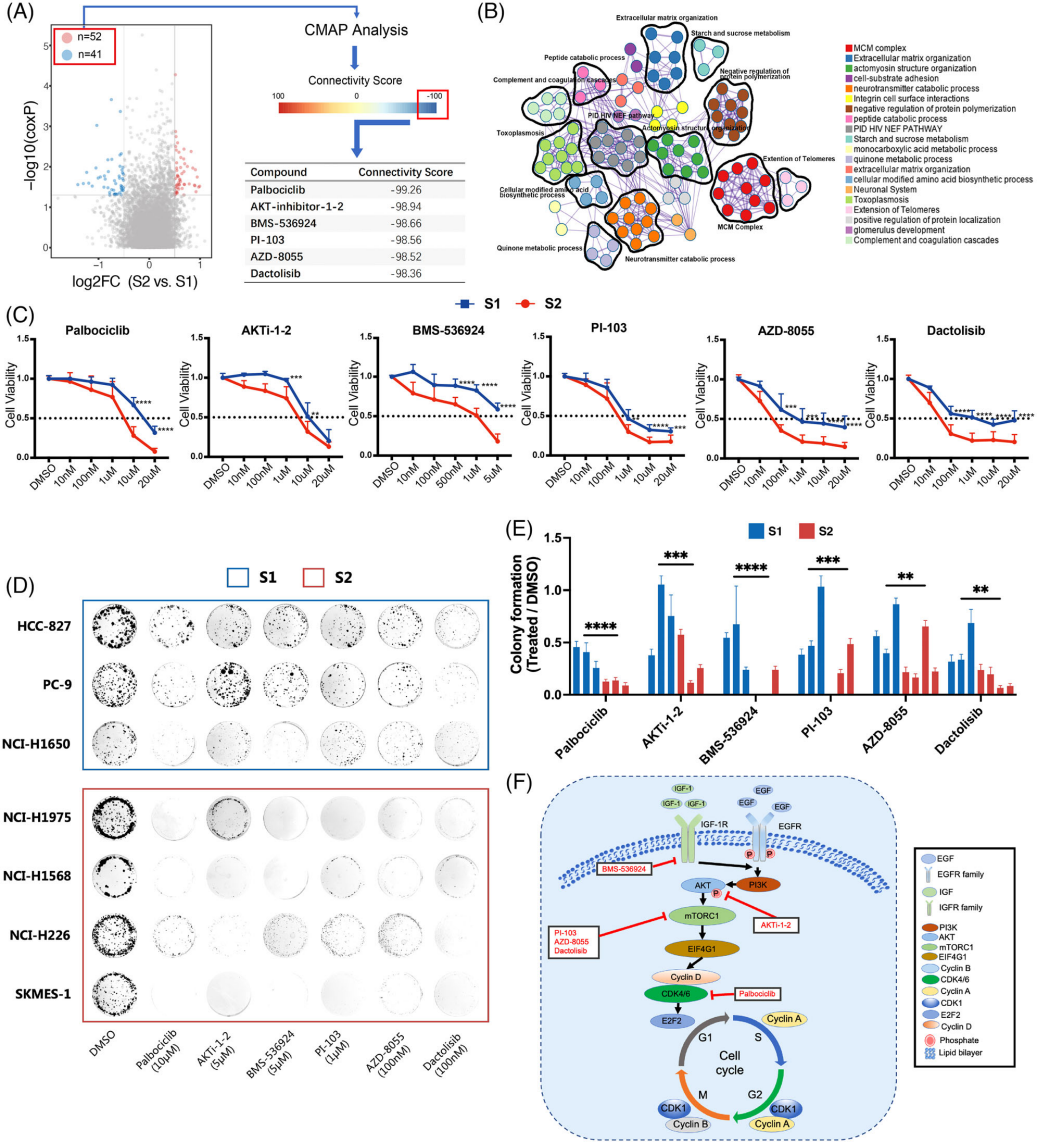
Proteomic analysis enables drug prediction and validation for subtype‐specific epidermal growth factor receptor (EGFR)‐mutant non‐small cell lung cancer (NSCLC) patients. (A) Scheme of connectivity map (CMAP)‐based drug prediction workflow. (B) Proteins that were selected as query signatures were applied to perform pathway and process enrichment in Metascape. (C) Cell viability of selected EGFR‐mutant NSCLC cell lines from S1 and S2 subtype treated with six candidate drugs at indicated concentration for 72 h. Representative result from three biological repeats was shown (mean ± SD). (D) Colony formation assays of seven cell lines treated with DMSO or six drugs as indicated. Representative data from three biological repeats were shown. Palbociclib, 10 μM; AKT‐inhibitor‐1‐2, 5 μM; BMS‐536924, 5 μM; PI‐103, 1 μM; AZD‐8055, 100 nM; Dactolisib, 100 nM. (E) Comparison of relative level of colony formation in S1 and S2 subtype (mean ± SD). (F) Scheme shows potential signalling pathways and drug targets specific for S2 subtype. *p*‐Values in (C and E) were calculated using unpaired Student's *t*‐test. *p < .05, **p < .01, ***p < .001, ****p < .0001.

In conclusion, our study classifies EGFR‐mutant NSCLC patients into two subtypes, S1 and S2, using multi‐omics data. Preliminary findings indicate a less favorable prognosis for S2 patients, characterized by an immune‐inflamed microenvironment and up‐regulation of cell cycle and mTOR signalling, which requires further validation in future studies. We also suggested the potential agents for such tumours (Figure 4F). Overall, our study enhances the potential for more precise diagnoses and development of more personalized treatment strategies, particularly for S2 patients.

## CONFLICT OF INTEREST STATEMENT

The authors have declared that no conflict of interest exists.

## Supporting information



Supporting Information

Supporting Information

## Data Availability

All data and materials within this study are publicly available in the TCGA (https://cancergenome.nih.gov/), CPTAC (https://cptac‐data‐portal.georgetown.edu/), Depmap (https://depmap.org/portal/) database, as well as the supplementary materials of original article.

## References

[1] Chen J , Yang H , Teo ASM , et al. Genomic landscape of lung adenocarcinoma in East Asians. Nat Genet. 2020;52:177‐186. 10.1038/s41588-019-0569-6 32015526

[2] Qiao M , Jiang T , Liu X , et al. Immune checkpoint inhibitors in EGFR‐mutated NSCLC: dusk or dawn? J Thorac Oncol. 2021;16(8):1267‐1288. 33915248 10.1016/j.jtho.2021.04.003

[3] Pan J , Cai X , Cao Z , et al. Osimertinib in the treatment of EGFR mutation‐positive advanced non‐small cell lung cancer: a meta‐analysis. Pharmacology. 2023;108:8‐16. 10.1159/000527321 36470213

[4] Yue D , Xu S , Wang Q , et al. Updated overall survival and exploratory analysis from randomized, phase II EVAN study of erlotinib versus vinorelbine plus cisplatin adjuvant therapy in stage IIIA epidermal growth factor receptor+ non‐small‐cell lung cancer. J Clin Oncol. 2022;40:3912‐3917. 10.1200/JCO.22.00428 36027483

[5] Wu Y‐L , Tsuboi M , He J , et al. Osimertinib in resected EGFR‐mutated non–small‐cell lung cancer. N Engl J Med. 2020;383:1711‐1723. 10.1056/nejmoa2027071 32955177

[6] Ramalingam SS , Vansteenkiste J , Planchard D , et al. Overall survival with osimertinib in untreated, EGFR‐mutated advanced NSCLC. N Engl J Med. 2020;382:41‐50. 10.1056/nejmoa1913662 31751012

[7] Chen Y‐J , Roumeliotis TI , Chang Y‐H , et al. Proteogenomics of non‐smoking lung cancer in East Asia delineates molecular signatures of pathogenesis and progression. Cell. 2020;182:226‐244.e17. 10.1016/j.cell.2020.06.012 32649875

[8] Gillette MA , Satpathy S , Cao S , et al. Proteogenomic characterization reveals therapeutic vulnerabilities in lung adenocarcinoma. Cell. 2020;182:200‐225.e35. 10.1016/j.cell.2020.06.013 32649874 PMC7373300

[9] Xu JY , Zhang C , Wang X , et al. Integrative proteomic characterization of human lung adenocarcinoma. Cell. 2020;182:245‐261.e17. 10.1016/j.cell.2020.05.043 32649877

[10] Chua KP , Teng YHF , Tan AC , et al. Integrative profiling of T790M‐negative EGFR‐mutated NSCLC reveals pervasive lineage transition and therapeutic opportunities. Clin Cancer Res. 2021;27:5939‐5950. 10.1158/1078-0432.CCR-20-4607 34261696 PMC9401458

