# Supplementary material for: Integrated analysis of EGFR mutated non‐small cell lung cancer reveals two distinct molecular subtypes
Source: Clin Transl Med. 2023 Oct 13;13(10):e1431. doi: 10.1002/ctm2.1431 (PMC10570769; doi:10.1002/ctm2.1431)
Supplement: Supplementary file 1 — Supporting Information [file CTM2-13-e1431-s002.docx]

**Methods and Materials**

**Publicly available datasets collection and processing**

We retrospectively collected the publicly available datasets of Multi-omics sequencing data and clinical annotations of NSCLC samples with detected EGFR mutations from Chen et al.[1], Xu et al.[2], Clinical Proteomic Tumor Analysis Consortium (CPTAC) lung adenocarcinoma database[3], The Cancer Genome Atlas-Lung Adenocarcinoma (TCGA-LUAD), and GIS023 cohort[4]. Tumors with nonsynonymous EGFR mutations, including frameshift, in-frame, missense, nonsense, and splice site mutations, were classified as EGFR-mutant tumors. Finally, our study enrolled 214 EGFR mutated NSCLC samples with available omics and clinical data (Table S1-4). In order to increase the sample size for our analysis, we integrated data from both the Chen and CPTAC datasets, both of which utilized the Hiseq 4000 platform from Illumina for RNA Sequencing (RNA-Seq). RNA-Seq data from both datasets were log2 transformed and quantile normalized. We combined these two cohorts to create a larger meta-cohort, which was performed using the "ComBat" function from the R package "sva" to remove any potential batch effects. TCGA-LUAD RNA-Seq dataset were downloaded from UCSC Xena website (﻿<https://gdc.xenahubs.net/>) in FPKM format, and was transformed into transcripts per kilobase million (TPM) format. Gene expression data and clinical information of GIS023 cohort were downloaded from ﻿OncoSG (https://src.gisapps.org/OncoSG/).

The proteomic and phosphoproteomic data were collected from Chen, CPTAC and Xu cohorts. In general, the global proteomics and phosphoproteimics of Chen and CPTAC cohorts were analyzed using TMT10-labeld LC/MS. Quantification of global proteome and phosphoproteome data was performed by Proteome Discoverer or Spectrum Mill in Chen and CPTAC cohorts, respectively. The relative abundances of proteomics and phosphoproteomics data were log2 transformed and zero centered to obtain a final, relative abundance values. The proteomics and phosphoproteomics of Xu cohort were subjected to Nano-LC-MS/MS analysis. Protein quantification were performed using iBAQ, a label-free quantification algorithm. The iBAQ values were calculated by Maxquant software and were quantile normalized and log2 transformed for further analysis.

**Data imputation**

KNN method of R package “impute” was applied for missing values imputation of RNA-Seq, proteomic and phosphoproteomic data. For RNA-seq and proteomic data, genes or proteins presented in at least 70% samples were reserved with the imputation parameters: k=5, rowmax=0.3, colmax=0.4. For phosphoproteomic data, phosphosites presented in at least 50% samples were reserved, with the imputation parameters: k=5, rowmax=0.5, colmax=0.4, in accordance to previous report[6].

**Unsupervised clustering of EGFR-mutant tumor samples**

The paired transcriptomics, proteomics and phosphoproteomics data of 59 tumor samples from Chen cohort were imputed and fused into a similarity matrix using the R package “CancerSubtypes” with default parameters. Using the similarity matrix as input, the unsupervised clustering was performed with the R package “ConsensusClusterPlus”[5] with the parameters: maxK=10, reps=500, clusterAlg= “spectralAlg”. The number of clusters was determined by the stable shape and maximum area of the consensus cumulative distribution function (CDF) curve with the clearest consensus matrix and the rapid decrease of average silhouette from k=2-4. To calculate NMI values for three data types, the function “rankFeatureByNMI” in the R package “SNFtools” were adopted using the default parameters. 3D projection of EGFR-mutant NSCLC clustering was performed by UMAP-based dimension reduction and visualized by “ploty” package.

**Variable selection analysis and subtype prediction**

Random forest algorithm was applied for the selection of subgroup specific signatures for EGFR-mutant subgroups using R package “VSURF” with number of trees set to 10,000 as previously described[7]. Selected mRNA or protein signature form interpretation step were used to build the prediction model. For subgroup prediction in EGFR-WT subgtoups, the 'predict' function in the R package 'VSURF' was applied, with the parameters 'type = class' and 'step = interp'. The subtypes of EGFR-mutant tumors in TCGA, CPTAC, and Xu cohorts were acquired by unsupervised clustering based on mRNA or proteomic signatures. As only transcriptomic data was available in TCGA-LUAD cohort, subgroup prediction was performed using mRNA signatures. CPTAC and Xu cohort were performed using protein signatures.

**Comparison of genomic alterations**

For genomic analysis, we obtained somatic mutation data as well as somatic copy number alteration (SCNA) segment data of CPTAC and TCGA-LUAD cohort. “ExtractSignatures” function from R package “maftools”, which based on Bayesian variant nonnegative matrix factorization, factorized the mutation portrait matrix into two nonnegative matrices “signatures” and “contributions”, where “signatures” represent mutational processes and “contributions” represent the corresponding mutational activities. The extracted mutational portrait of EGFR-mutant NSCLC was compared and annotated by cosine similarity analysis against the Catalogue of Somatic Mutations in Cancer (COSMIC V3). The 96 types single nucleotide variants of EGFR-mutant NSCLC landscape were profiled by Lego plot using R package “rgl”. We utilized GISTIC2 version 2.0.23 to determine gene-level copy number values and identify significant copy number alterations in the cohort[8].GISTIC2 generated arm level and focal level SCNAs for the cohort with G-Score and FDR-Q value indicating the significance and strength of the identified SCNAs. The aneuploidy scores and immune and cell-cycle signature scores of TCGA samples were determined and compiled from prior research[9].

**Pathway analysis**

To examine the differences in biological processes among different EGFR mutant subgroups, we conducted single sample Gene Set Enrichment Analysis (ssGSEA) at mRNA level using Hallmark gene set (from the MsigDB database v7.1) and PTM Signature Enrichment Analysis (PTM-SEA) at phosphoprotein level, using the PTM signatures database (PTMsigDB) [10]. GSEA analysis were also conducted using mRNA data and imputed protein data against Hallmark and C2 WikiPathways gene set with the R package "clusterProfiler"[11, 12]. Pathways with FDR <0.05 were considered significantly regulated. PTM signature database (PTMsigDB, v1.91) was downloaded from ﻿<http://prot-shiny-vm.broadinstitute.org:3838/ptmsigdb-app/>, using uniport id and phosphosite as primary identifier. We used the web portal of PTM-SEA in GenePattern (<https://cloud.genepattern.org/>), with the following parameters: weight:0.75; statistic: “area.under.RES”; output.score.type: “NES”; nperm: 1000; min.overlap: 5; correl.type: “z.score”.

**KSEA analysis**

To investigate different enriched kinases in each subgroup, we used the web portal of KSEA App (https://casecpb.shinyapps.io/ksea/) to conduct Kinase-substrate enrichment analysis (KSEA) [13] on phosphosite data with a p-value cutoff of <0.05 and a minimum substrate count of 1.

**Potential functionally important phosphorylation analysis**

Functionally annotated phosphosites (ON_PROCESS, induced or inhibited) were retrieved from PhosphoSitePlus database to identify functionally important phosphorylation in each subtype. All the statistically significantly differential phosphosites among two subtypes (Wilcoxon test, p<0.05) were calculated.

**Subset specific genetic dependency analysis**

The subtype specific cancer-dependent genes were analyzed using genetic dependency of CRISPR screening dataset from DepMap database (﻿<https://depmap.org/portal/>). The subtype specific cancer-dependent genes were selected according to 1) The mean dependency score difference of a gene between two subtypes was less than -0.35; 2) For each subtype, the dependency scores were significantly different between two subtypes (Wilcoxon rank-sum test, FDR<0.05); 3) Genes with mean dependency score <-0.5 in one subtype.

**Cell lines and culture conditions and reagents**

Available clinical annotations, expression profile and somatic mutation data of human NSCLC cell lines were obtained from the Cancer Cell Line Encyclopedia (CCLE) project of the Broad Institute. Cell lines with non-silent EGFR mutations (N=20) were included in this study. Cell lines were classified into subgroups using mRNA subgroup signatures. For experimental validation, HCC827, NCIH1650, NCIH1975, SKMES1, NCIH226 and NCIH1568 were purchased from ATCC; PC9 was purchased from National Collection of Authenticated Cell Cultures. All cells are cultured in RPMI-1640 medium (Gibco) supplemented with 10% fetal bovine serum (Gibco), penicillin (100mg/mL) and streptomycin (100mg/mL). All cells were maintained at 37℃ under the humidified 5% CO_2_ atmosphere. Palbociclib (HY-50767), AKT-inhibitor-1-2 (HY-10355), BMS-536924 (HY-10262), PI-103 (HY-10115), AZD-8055 (HY-10422) and Dactolisib (HY-50673) were purchased from MedChem Express.

**Western blotting**

For western blotting, the primary antibodies against the following proteins were used: mTOR (PTM-BIO Cat# PTM-6594, RRID: AB_2927749), p-mTOR (Cell Signaling Technology Cat# 5536, RRID:AB_10691552), DEPTOR (Proteintech Cat# 20985-1-AP, RRID:AB_11182391), 4EBP1 (Cell Signaling Technology Cat# 9644, RRID:AB_2097841), p-4EBP1 (Cell Signaling Technology Cat# 2855, RRID:AB_560835), EIF4G1 (Cell Signaling Technology Cat# 2858, RRID:AB_2095745), p-EIF4G1 (Cell Signaling Technology Cat# 2441, RRID:AB_2277632), β-actin (Proteintech Cat# 20536-1-AP, RRID:AB_10700003). Briefly, cells were lysed in RIPA lysis buffer. The protein concentration of cell lysates was determined by BCA protein assay kit. Up to 50ng/mL proteins were resolved by SDS-polyacrylamide gels and transferred to polyvinylidene fluoride membranes. The membrane was blocked with 5% skimmed milk powder diluted with Tris-buffered saline Tween-20 for 1 hour and incubated with corresponding primary antibodies at 4℃ overnight. The membranes were then washed and incubated with horseradish peroxidase-conjugated secondary antibodies (Cell Signaling Technology Cat# 7074, RRID:AB_2099233; Cell Signaling Technology Cat# 7076, RRID:AB_330924). ECL chemiluminescent detection reagent (WBKLS, Millipore) was applied for bands visualization.

**Dissecting the cellular components in the TME of EGFR mutant NSCLC**

To dissect the cellular components in the TME of EGFR mutant NSCLC, we utilized the xCell algorithm[14] based on bulk RNA-seq datasets. We prepared gene expression profiles using standard annotation files, and processed the data using the “deconvo_tme” function in the “IOBR” R package[15], with the parameters: method = “xcell”. The algorithm was run using the xCell signatures.

**TIDE analysis**

﻿Tumor Immune Dysfunction and Exclusion (TIDE) is an algorithm proposed by Jiang et al. to predict response to immune checkpoint blockade via modeling T cell dysfunction and exclusion in TME[16]. Tumors with higher TIDE score suggests occurrence of immune evasion, and lower response rate to ICB treatment. We utilized TIDE algorithm to predict response to ICB treatment in sample cohort based on transcriptomic data. Transcriptome data was processed according to instructions, and data was uploaded to the TIDE web portal (http://tide.dfci.harvard.edu/). Patients with TIDE score lower than zero were considered as responder to ICB treatment.

**CMAP based drug prediction**

Connectivity Map (CMAP) is a comprehensive resource that uses functional perturbations in cultured human cells, along with gene expression data, to identify connections between genes, drugs, and diseases[17]. To predict candidate drugs specific for S2 subgroup, we selected the proteins that meet the following criteria as query signature: (1) The protein expression is upregulated or downregulated in S2 subgroup (log2FC>0.5 or <-0.5; p<0.05); and (2) The protein expression was significantly correlated with progression-free survival (PFS, as determined by Cox analysis with a p-value <0.05). We identified perturbagens with the highest negative connectivity scores as potential drugs for patients in the S2 subgroup, as a high negative score indicated that the perturbagen reversed the expression of the query signature.

**Metascape analysis**

Metascape is a web-based portal that provides a comprehensive resource for annotating and analyzing pathways in order to understand the molecular mechanisms underlying a biological system within OMICs databases[18]. We used the query signature as input to Metascape portal followed by instructions with the cutoff of p<0.05 to investigate the biological process represented by the query signature. To investigate the biological processes represented by the query signature, we used the query signature as input to the Metascape portal, following the provided instructions, and set a p-value cutoff of <0.05.

**Cell viability assay**

Cells were seeded in 96-well plates at a density of 10,000 cells per 100μL per well. The cells were cultured for 12 hours for adhesion and then the culture medium was changed to one containing different drug concentrations. After 72 hours of continuous culture, 10μL of CCK8 reagent (40203ES80, Yeasen) was added to each well and incubated for 2 hours. The absorbance of each sample was then measured at 450nm.

**Colony formation assay**

Cell lines to be tested were seeded in 6-well plate (500-800 cells/well). The cells were cultured for 12 hours for adhesion and then the culture medium was changed to one containing different drug concentrations. After continuous culture for 14 days, cells were rinsed with cold phosphate-buffered saline (PBS), and fixed by methanol at 4℃ for 20 minutes, followed by staining with Giemsa. The colony were then photographed. Image J software (﻿US National Institutes of Health, Bethesda, MD, USA) were applied to counting the colony number.

**Statistical analysis**

In this study, we applied R version 4.1.3 for statistical analyses. Quantitative data were analyzed using Student's t-test for normally distributed variables and the Wilcoxon rank-sum test for non-normally distributed variables. To analyze contingency tables, we applied the chi-square test and Fisher’s exact test based on specific conditions. Kaplan-Meier survival analysis were adopted to exam the prognosis of different subgroups. Uni-variate Cox analysis was applied to investigate the association between protein expression and prognosis. All comparisons were two-sided with an alpha level of 0.05. For multiple hypothesis testing, to control the false discovery rate (FDR), the Benjamini-Hochberg method was applied.

**Limitations**

While we utilized multi-omics data from various patient cohorts to develop molecular subtypes of EGFR-mutant NSCLC, the total sample size across these cohorts remains relatively small. The limited sample size might restrict the generalizability of our findings, and larger independent cohorts would be valuable for validation of these subtypes in future studies. Moreover, EGFR-TKIs are recognized as significantly impacting patient outcomes, hence the absence of a comprehensive analysis incorporating treatment data, such as chemotherapy and immunotherapy in late-line settings, constrains our ability to adjust for this influential factor. Finally, while our analysis suggests potential effectiveness of drugs targeting the cell cycle and mTOR signaling pathway for the S2 subtype, individual patient responses may vary considerably. Further in vivo validations are warranted to refine these subtype-specific therapeutic strategies.

**References**

1. Chen Y-J, Roumeliotis TI, Chang Y-H, et al (2020) Proteogenomics of Non-smoking Lung Cancer in East Asia Delineates Molecular Signatures of Pathogenesis and Progression. Cell 182:226-244.e17. https://doi.org/10.1016/j.cell.2020.06.012

2. Xu JY, Zhang C, Wang X, et al (2020) Integrative Proteomic Characterization of Human Lung Adenocarcinoma. Cell 182:245-261.e17. https://doi.org/10.1016/j.cell.2020.05.043

3. Gillette MA, Satpathy S, Cao S, et al (2020) Proteogenomic Characterization Reveals Therapeutic Vulnerabilities in Lung Adenocarcinoma. Cell 182:200-225.e35. https://doi.org/10.1016/j.cell.2020.06.013

4. Chua KP, Teng YHF, Tan AC, et al (2021) Integrative profiling of T790M-negative EGFR-mutated NSCLC reveals pervasive lineage transition and therapeutic opportunities. Clin Cancer Res 27:5939–5950. https://doi.org/10.1158/1078-0432.CCR-20-4607

5. Chong W, Zhu X, Ren H, et al (2022) Integrated multi-omics characterization of KRAS mutant colorectal cancer. Theranostics 12:5138–5154. https://doi.org/10.7150/thno.73089

6. Gillette MA, Clauser KR, Wang P, et al (2016) Proteogenomics connects somatic mutations to signalling in breast cancer. Nature 534:55–62. https://doi.org/10.1038/nature18003

7. Liu Z, Liu Y, Qian L, et al (2021) A proteomic and phosphoproteomic landscape of KRAS mutant cancers identifies combination therapies. Mol Cell 81:4076-4090.e8. https://doi.org/10.1016/j.molcel.2021.07.021

8. Mermel CH, Schumacher SE, Hill B, et al (2011) GISTIC2.0 facilitates sensitive and confident localization of the targets of focal somatic copy-number alteration in human cancers. Genome Biol 12:R41. https://doi.org/10.1186/gb-2011-12-4-r41

9. Davoli T, Uno H, Wooten EC, Elledge SJ (2017) Tumor aneuploidy correlates with markers of immune evasion and with reduced response to immunotherapy. Science (80- ) 355:. https://doi.org/10.1126/science.aaf8399

10. Krug K, Mertins P, Zhang B, et al (2019) A Curated Resource for Phosphosite-specific Signature Analysis. Mol Cell Proteomics 18:576–593. https://doi.org/10.1074/mcp.TIR118.000943

11. Yu G, Wang LG, Han Y, He QY (2012) ClusterProfiler: An R package for comparing biological themes among gene clusters. Omi A J Integr Biol 16:284–287. https://doi.org/10.1089/omi.2011.0118

12. Wu T, Hu E, Xu S, et al (2021) clusterProfiler 4.0: A universal enrichment tool for interpreting omics data. Innov 2:100141. https://doi.org/10.1016/j.xinn.2021.100141

13. Wiredja DD, Koyutürk M, Chance MR (2017) The KSEA App: a web-based tool for kinase activity inference from quantitative phosphoproteomics. Bioinformatics 33:3489–3491. https://doi.org/10.1093/bioinformatics/btx415

14. Aran D, Hu Z, Butte AJ (2017) xCell: Digitally portraying the tissue cellular heterogeneity landscape. Genome Biol 18:1–14. https://doi.org/10.1186/s13059-017-1349-1

15. Zeng D, Ye Z, Shen R, et al (2021) IOBR: Multi-Omics Immuno-Oncology Biological Research to Decode Tumor Microenvironment and Signatures. Front Immunol 12:1–9. https://doi.org/10.3389/fimmu.2021.687975

16. Jiang P, Gu S, Pan D, et al (2018) Signatures of T cell dysfunction and exclusion predict cancer immunotherapy response. Nat Med 24:1550–1558. https://doi.org/10.1038/s41591-018-0136-1

17. Subramanian A, Narayan R, Corsello SM, et al (2017) A Next Generation Connectivity Map: L1000 Platform and the First 1,000,000 Profiles. Cell 171:1437-1452.e17. https://doi.org/10.1016/j.cell.2017.10.049

18. Zhou Y, Zhou B, Pache L, et al (2019) Metascape provides a biologist-oriented resource for the analysis of systems-level datasets. Nat Commun 10:. https://doi.org/10.1038/s41467-019-09234-6

**Supplementary Figures**


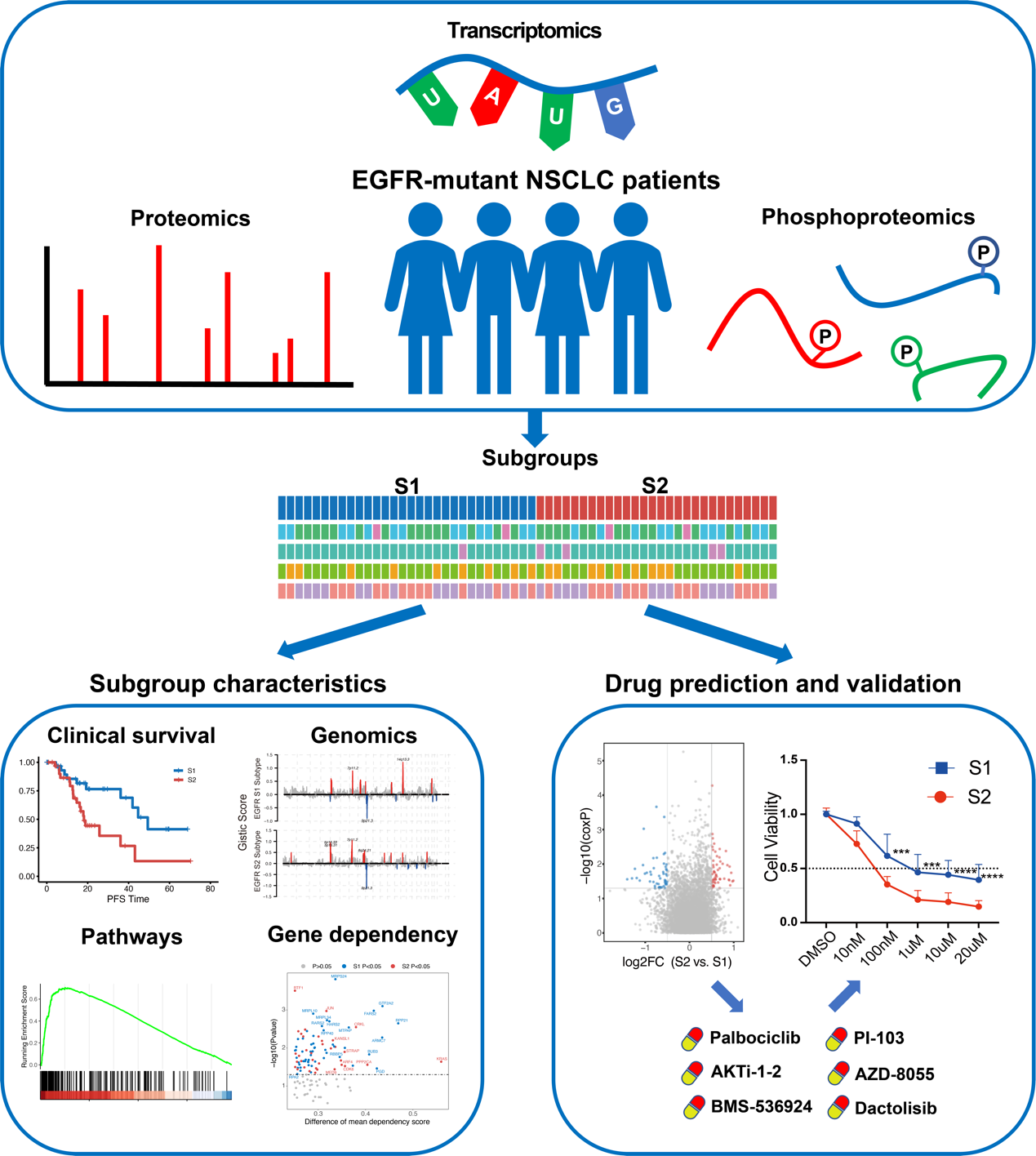


**Figure S1. Scheme of the integrated analysis of multi-omics data of EGFR mutant NSCLC tumors**


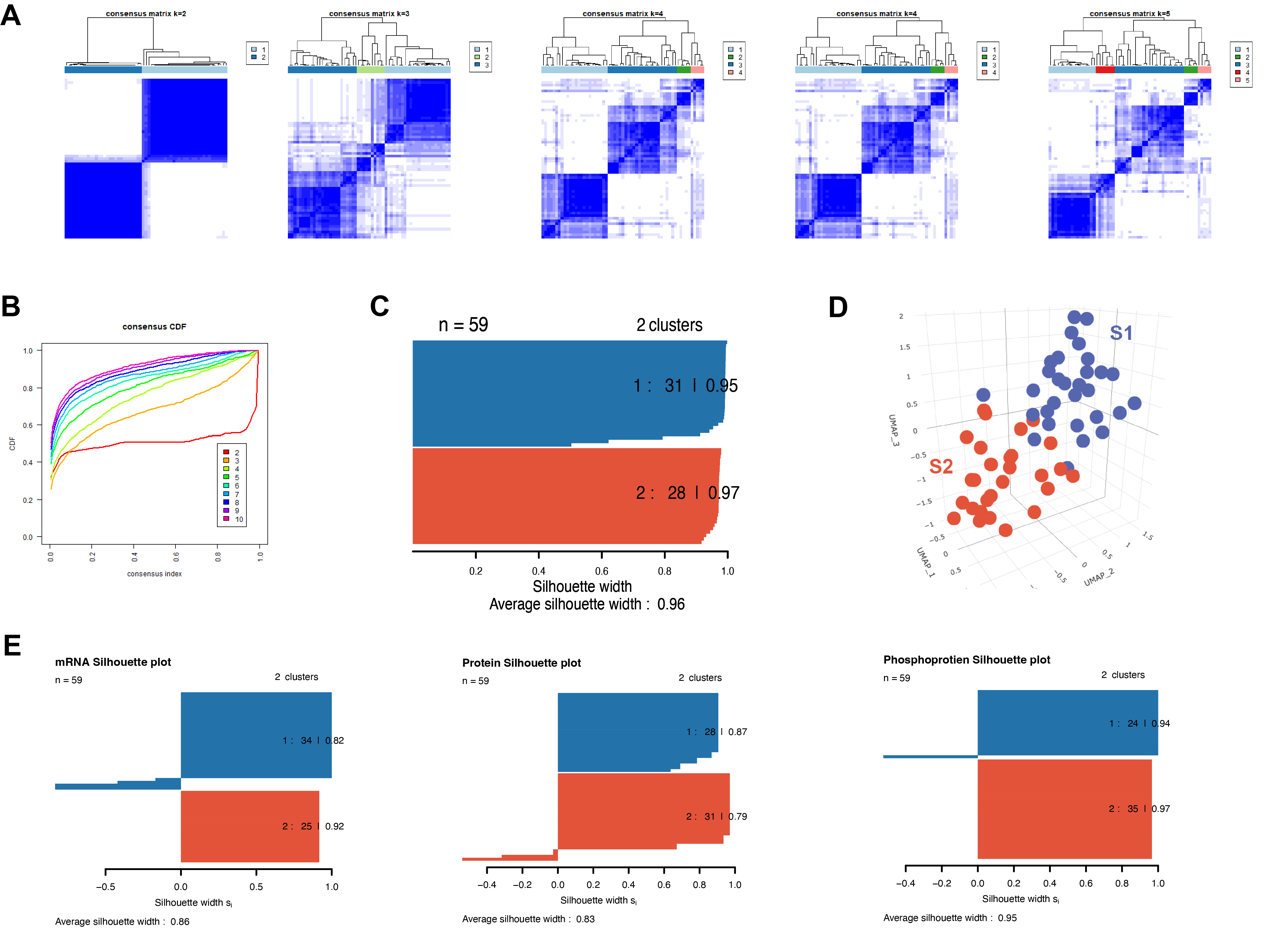


**Figure S2. Integrative subtypeing of EGFR-mutant NSCLC tumors based on multi-omics data.** (A) Consensus matrix of unsupervised clustering of Chen cohort based on integrated multiomics data with cluster numbers from 2 to 6. (B) The consensus CDF of unsupervised clustering based on multi-omics data. (C) The silhouette width of unsupervised clustering based on SNF method in integrated omics data when k=2. (D) 3D UMAP projections of EGFR-mutant NSCLC tumors per different subtypes. (E) The silhouette width of unsupervised clustering based on single layer of omic data with SNF method when k=2.


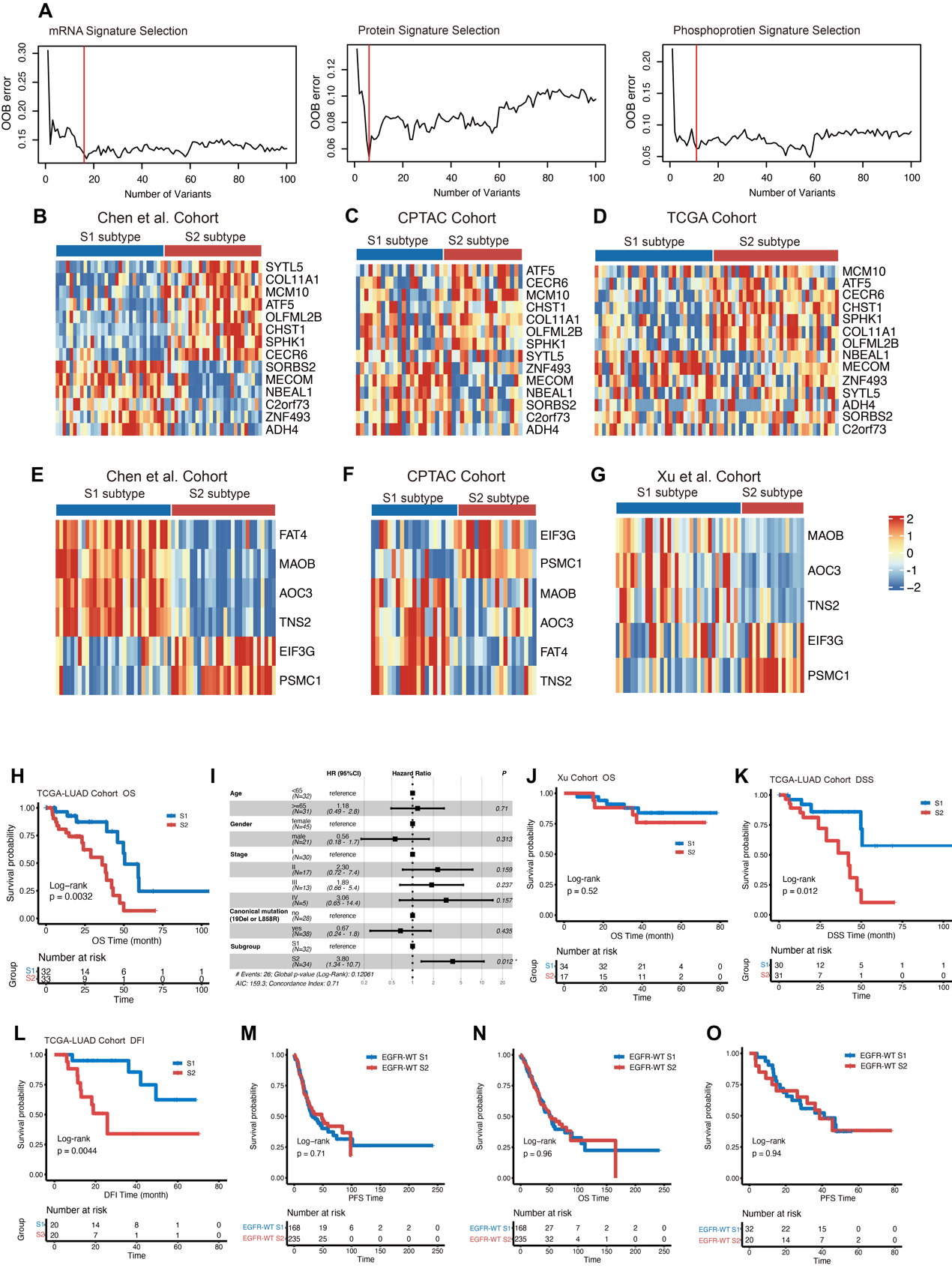


**Figure S3. Identification of molecular signatures for EGFR-mutant subtypes enables subtyping for EGFR mutant or WT tumor samples.** (A) The out of bag (OOB) error rate in signature selection for mRNA, protein and phosphoprotein using random forest. (B-D) Heatmap of signature mRNAs in EGFR mutant tumors of Chen, CPTAC and TCGA cohort. (E-G) Heatmap of signature proteins in EGFR mutant tumors of Chen, CPTAC and Xu cohort. (H) OS of the two EGFR-mutant subtypes in TCGA cohort using Kaplan-Meier analysis. (I) The association between the two EGFR-mutant subtypes and OS in TCGA cohort after being adjusted for age, gender, stage, mutation type using multivariate Cox model. (J) OS of the two EGFR-mutant subtypes in Xu cohort using Kaplan-Meier analysis. (K) DSS of the two EGFR-mutant subtypes in TCGA cohort using Kaplan-Meier analysis. (L) DFI of the two EGFR-mutant subtypes in TCGA cohort using Kaplan-Meier analysis. (M-O) PFS and OS of the two EGFR-WT subtypes in TCGA cohort using Kaplan-Meier analysis. (L) PFS of the two EGFR-WT subtypes in Xu cohort using Kaplan-Meier analysis. P values in (H, J-L) were from log rank test.


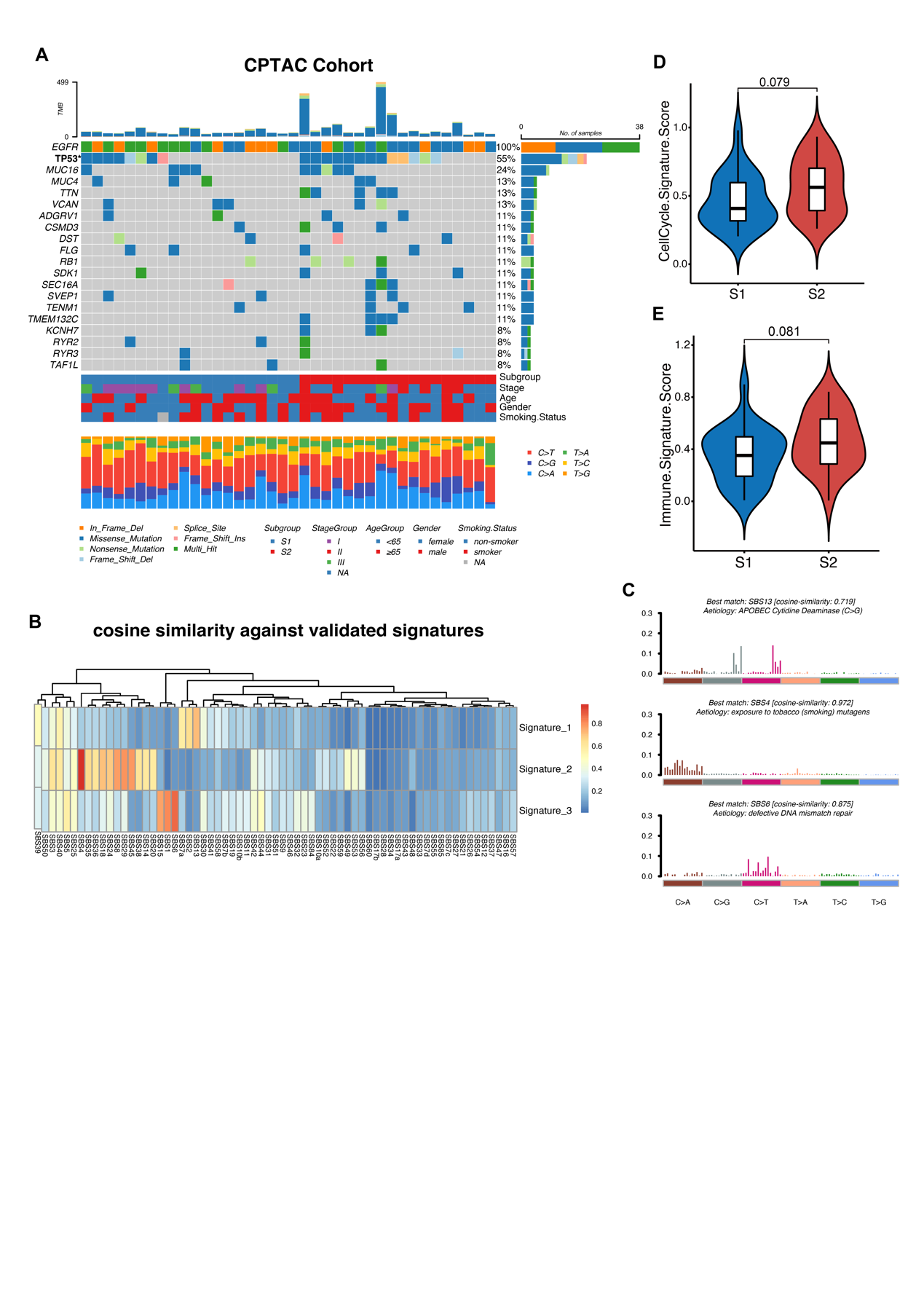


**Figure S4. Tumor genomic variations in two EGFR-mutant NSCLC subtypes.** (A) Mutational landscape of EGFR-mutant NSCLC in different subtypes of CPTAC cohort. (B) Heatmap shows the cosine similarity analysis of extracted 3 mutational signatures against single base substitution (SBS) signatures from Catalogue of Somatic Mutations in Cancer. (C) Mutational activities of extracted mutational signatures (SBS13, SBS4 and SBS6). (D) Comparison of Cell Cycle signature score in different subtypes of EGFR mutant NSCLC tumors (p=0.079, Wilcoxon test). (E) Comparison of Immune signature score in different subtypes of EGFR mutant NSCLC tumors (p=0.081, Wilcoxon test).


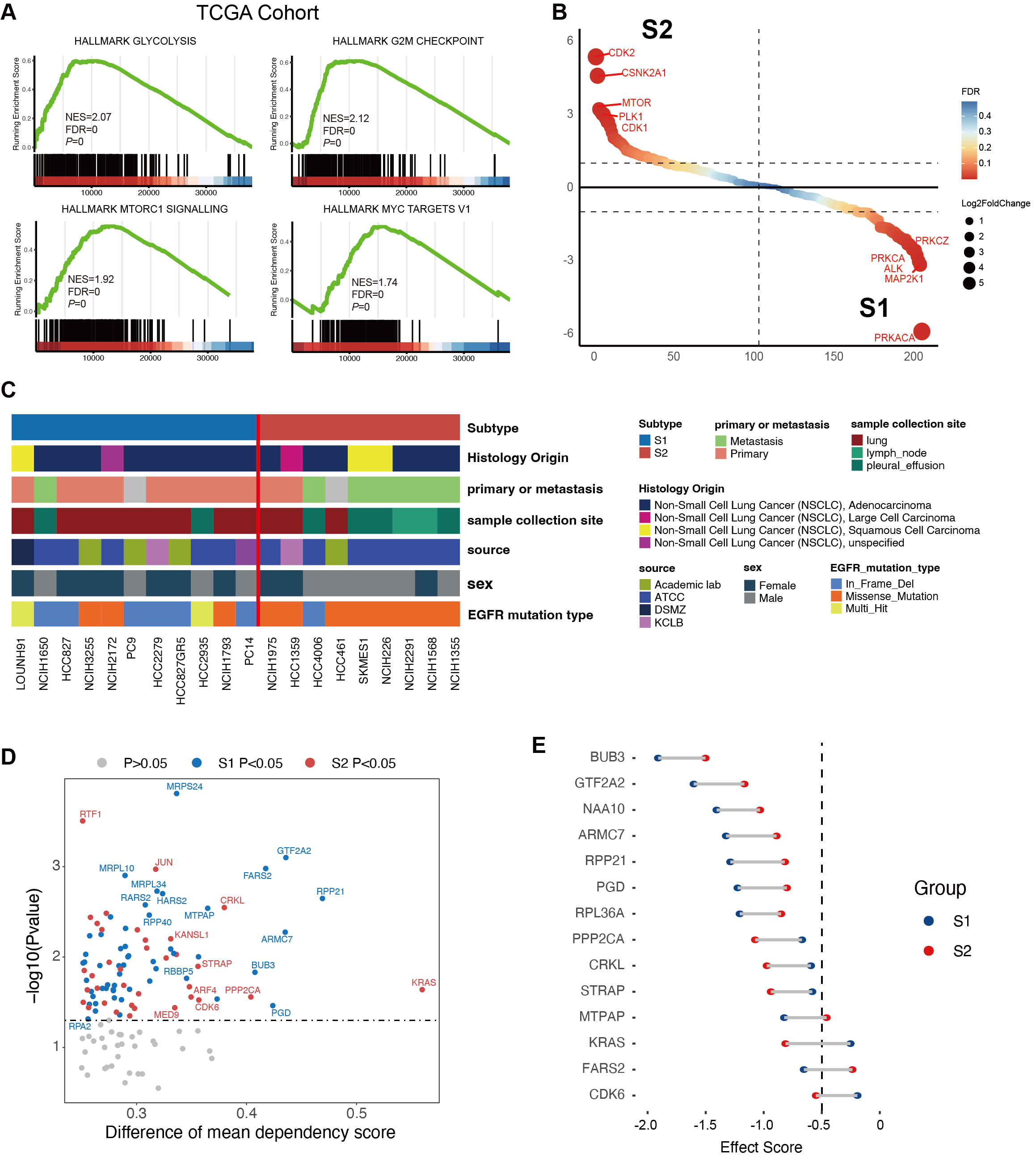


**Figure S5. Characterization of distinct biological pathways in EGFR-mutant subtypes.** (A) GSEA enrichment plots of representative pathways of S2 subtype in TCGA cohort. (B) Top 5 enriched kinases in each EGFR-mutant subtype revealed by KSEA. Kinases at lower-right corner are enriched in S1 subtype, upper-left corner are enriched in S2 subtype. (C) Subtyping of EGFR-mutant NSCLC cell lines with relevant clinical feature annotations. (D) Differences of gene dependency between S1 and S2 subtypes. (E) Mean dependency score of subtype specific genes (mean dependency score difference < -0.35, Wilcoxon test, p<0.05).


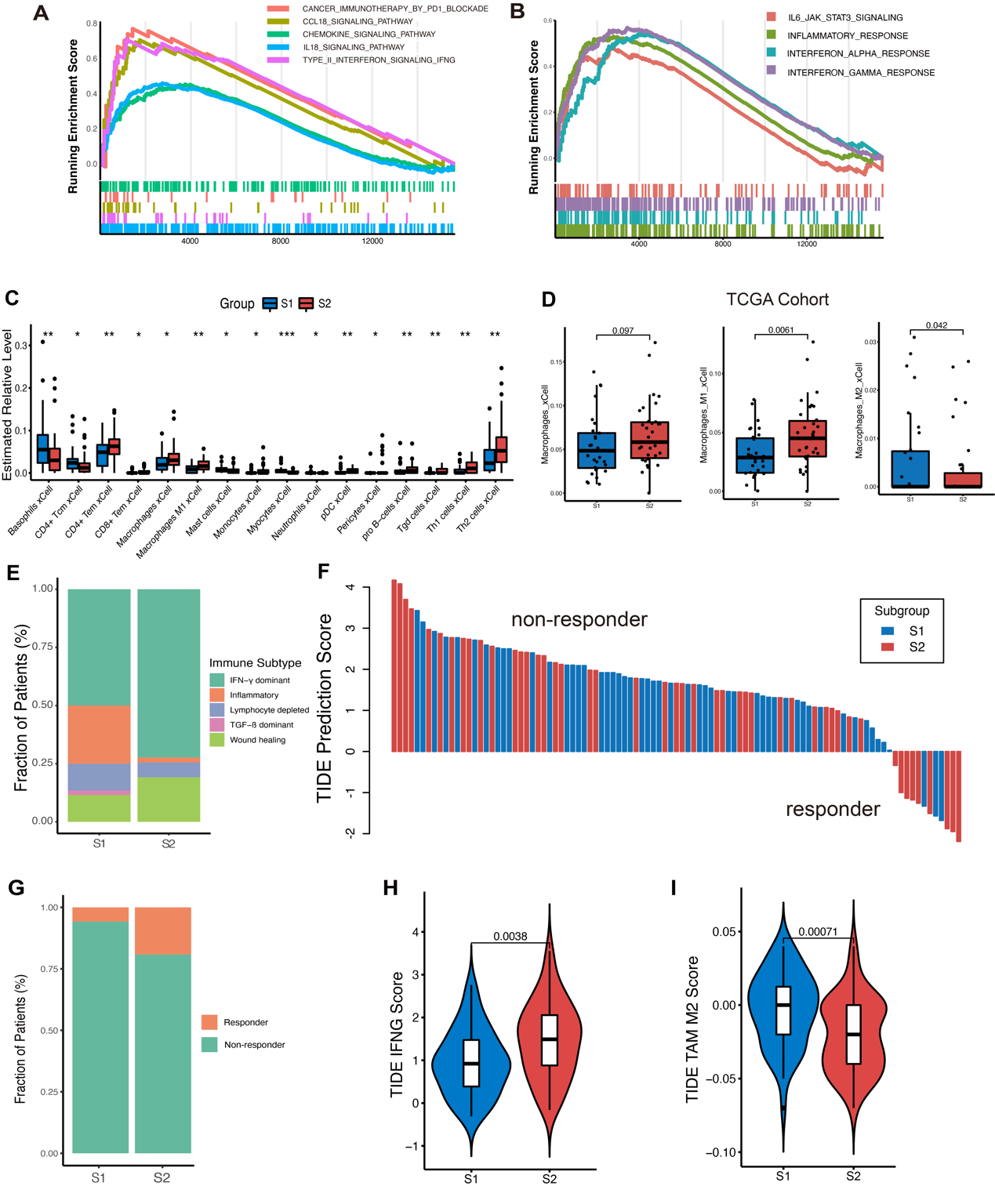


**Figure S6. Distinct tumor immune microenvironment (TIME) in two EGFR-mutant subtypes.** (A) GSEA enrichment plot shows enriched immune-related pathways in WikiPathways gene sets in S2 subtype. (B) GSEA enrichment plots shows enriched immune-related pathways in hallmark gene set in S2 subtype. (C) Comparison of TME cellular components in different EGFR-mutant subtypes using xCell algorithm in Chen-CPTAC meta-cohort. *p<0.05, **p<0.01 and ***p<0.001. (D) Comparison of cellular proportion of Macrophages, Macrophage M1 and Macrophage M2 in different EGFR-mutant subtypes of TCGA cohort. (E) Comparison of the proportion of different immune subtypes in different EGFR-mutant subtypes in Chen-CPTAC meta-cohort (p=0.0082, Chi-square test). (F) Waterfall plot shows the TIDE prediction score in Chen-CPTAC meta-cohort. Patients from S1 subtype are represented in blue; patients from S2 subtype are represented in red. (G) Comparison of proportion of predicted responders to ICI therapy in different EGFR-mutant subtypes in Chen-CPTAC meta-cohort (p=0.083, Chi-square test). (H-I) Comparison of Interferon-gamma (IFNG) score and TAM M2 score in different EGFR-mutant subtypes (p=0.0038 and 0.00071 respectively, Wilcoxon test).


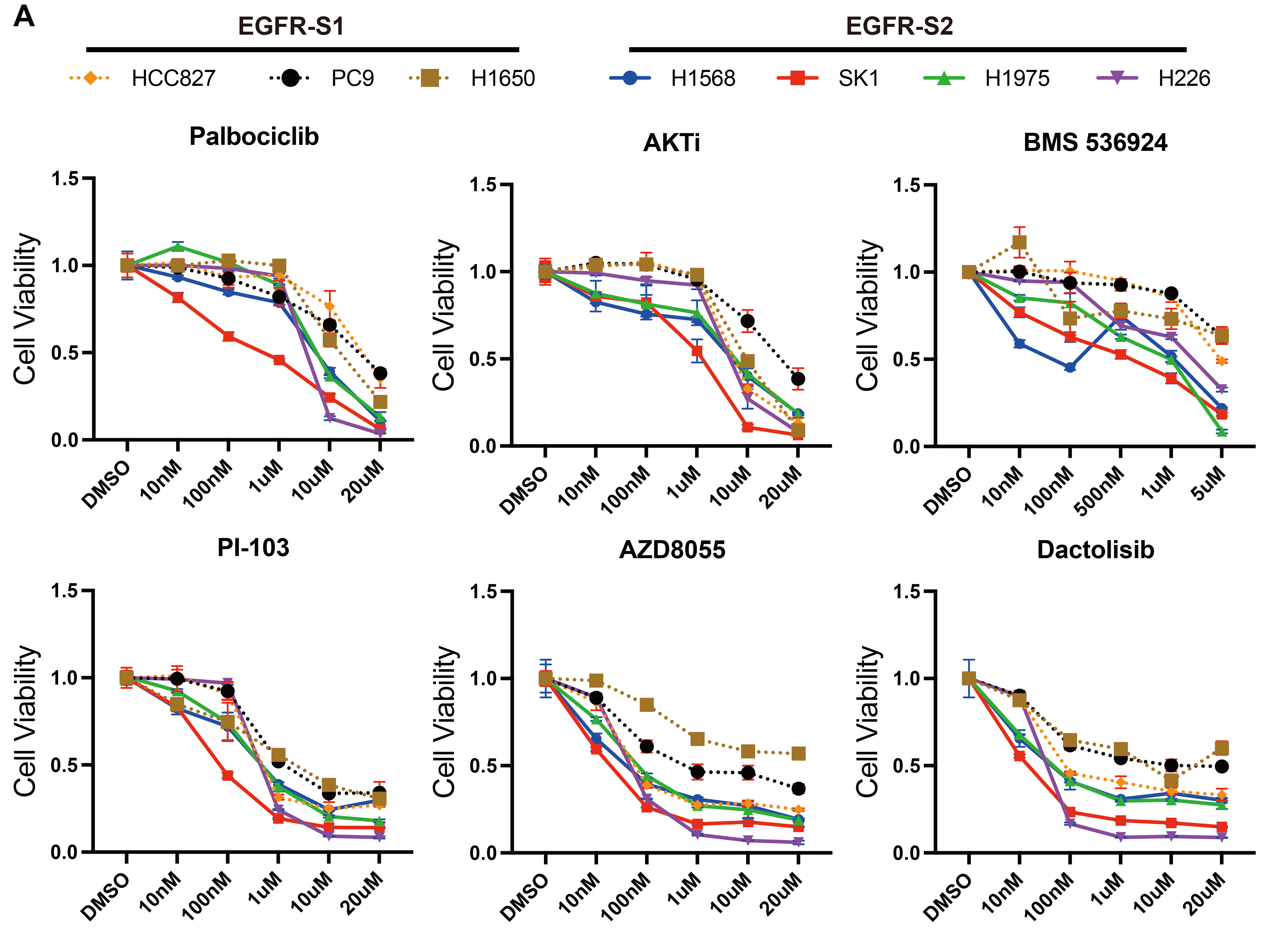


**Figure S7. Subtype specific therapeutic vulnerabilities in EGFR mutant NSCLC.** (C) Detailed result of cell viability of selected EGFR-mutant NSCLC cell lines from S1 and S2 subtype treated with six candidate drugs at indicated concentration for 72 hours. Representative result from three biological repeats were shown (mean ± SD).

﻿**Full unedited gel for Figure 3G**


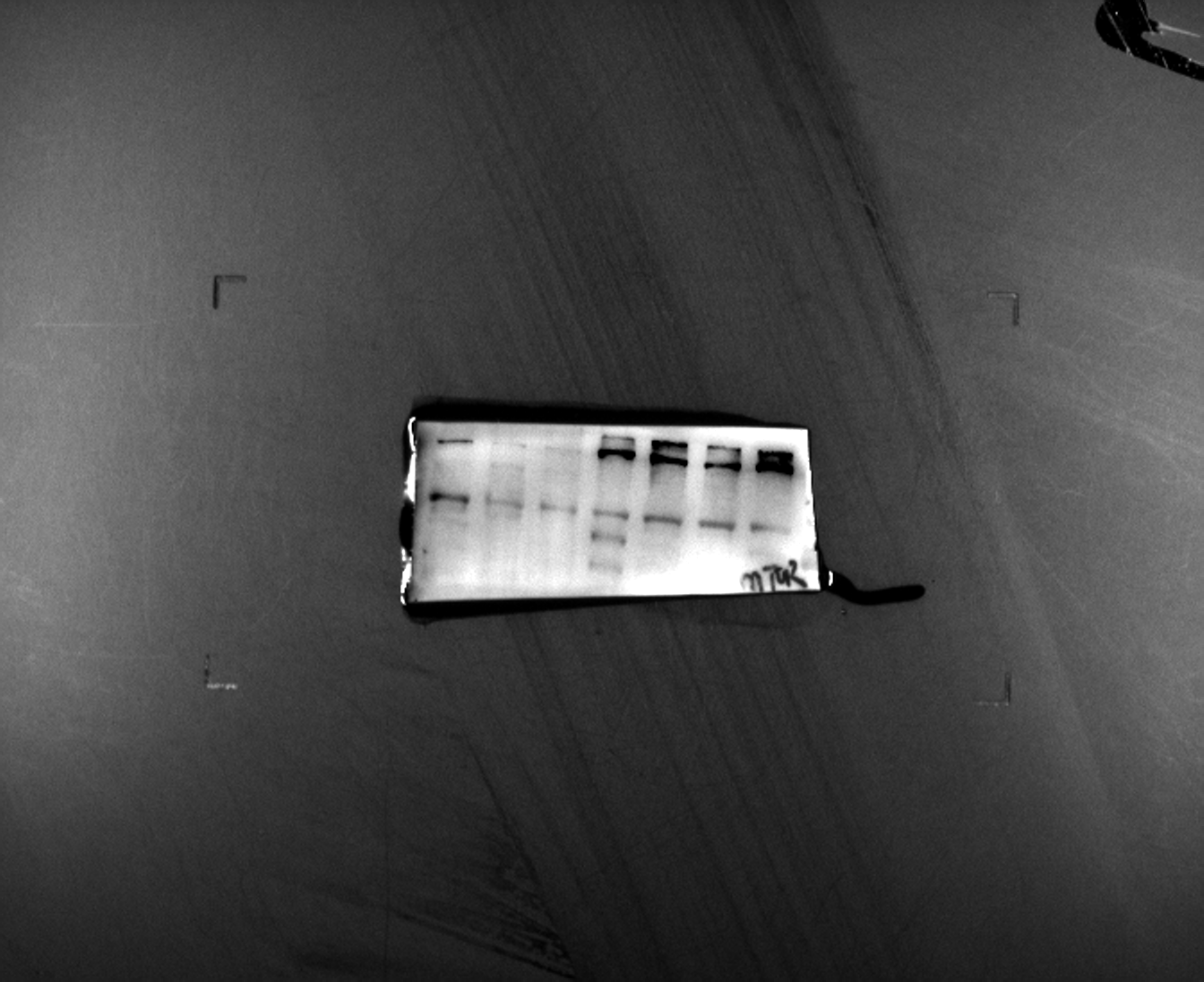

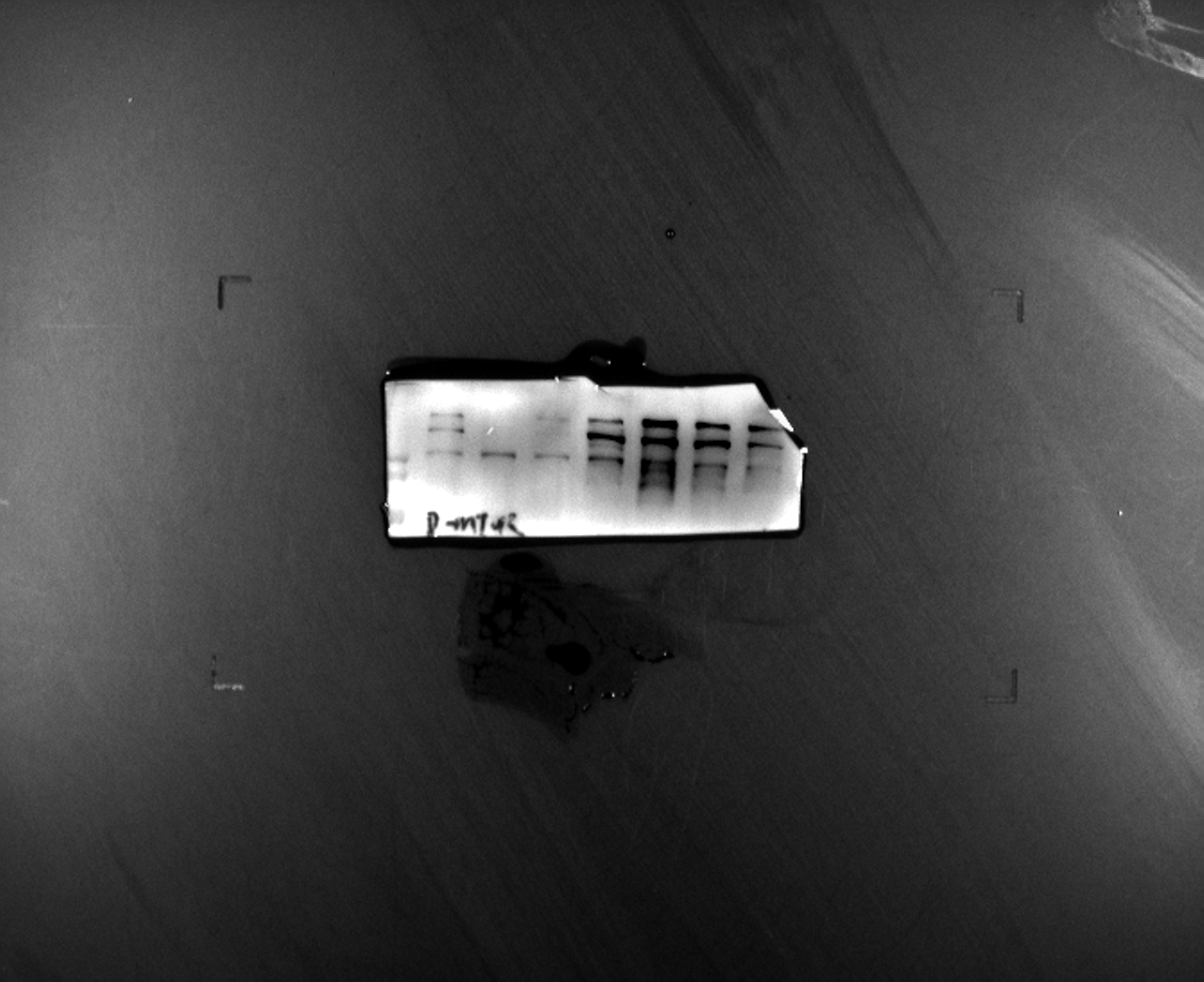

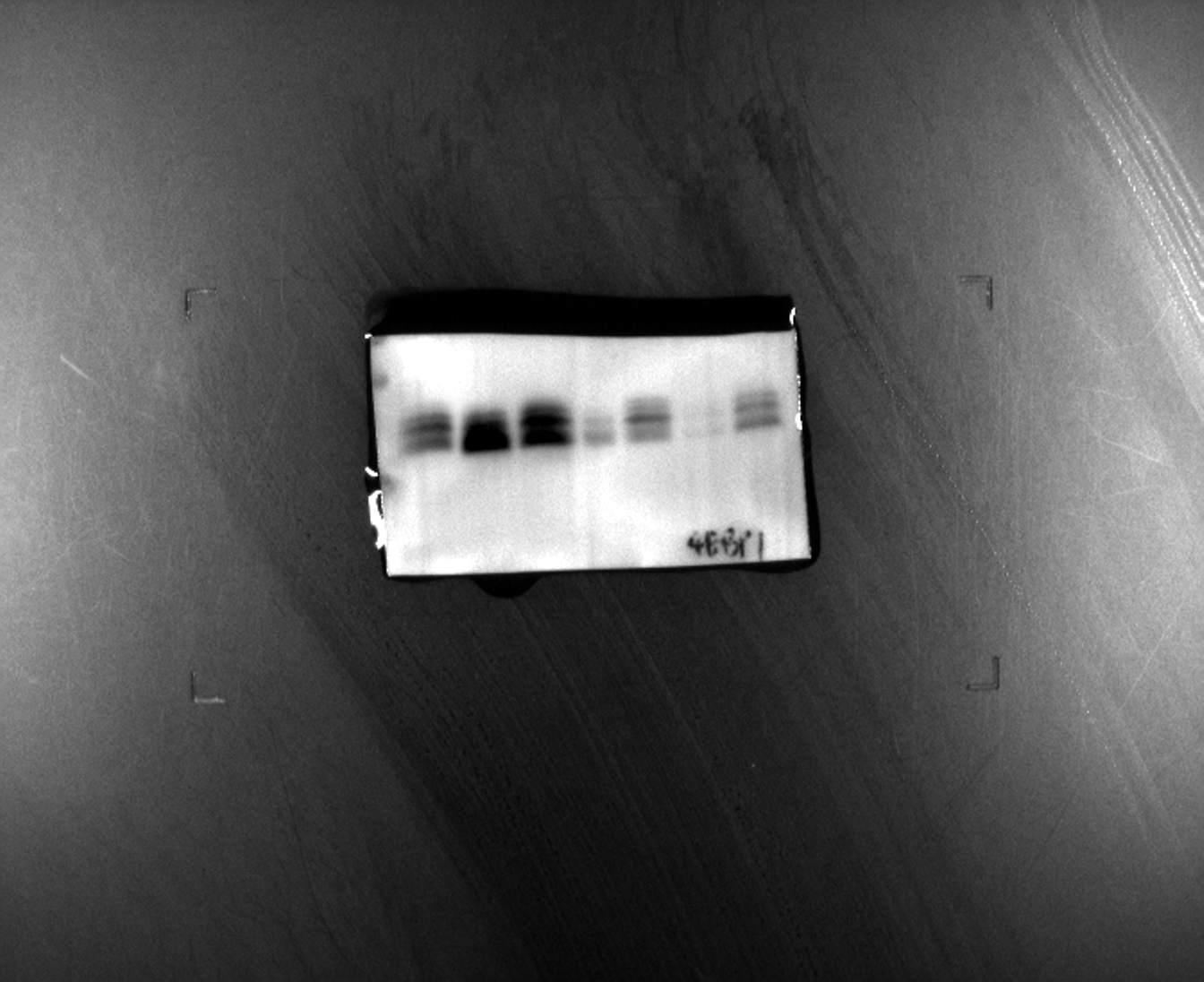

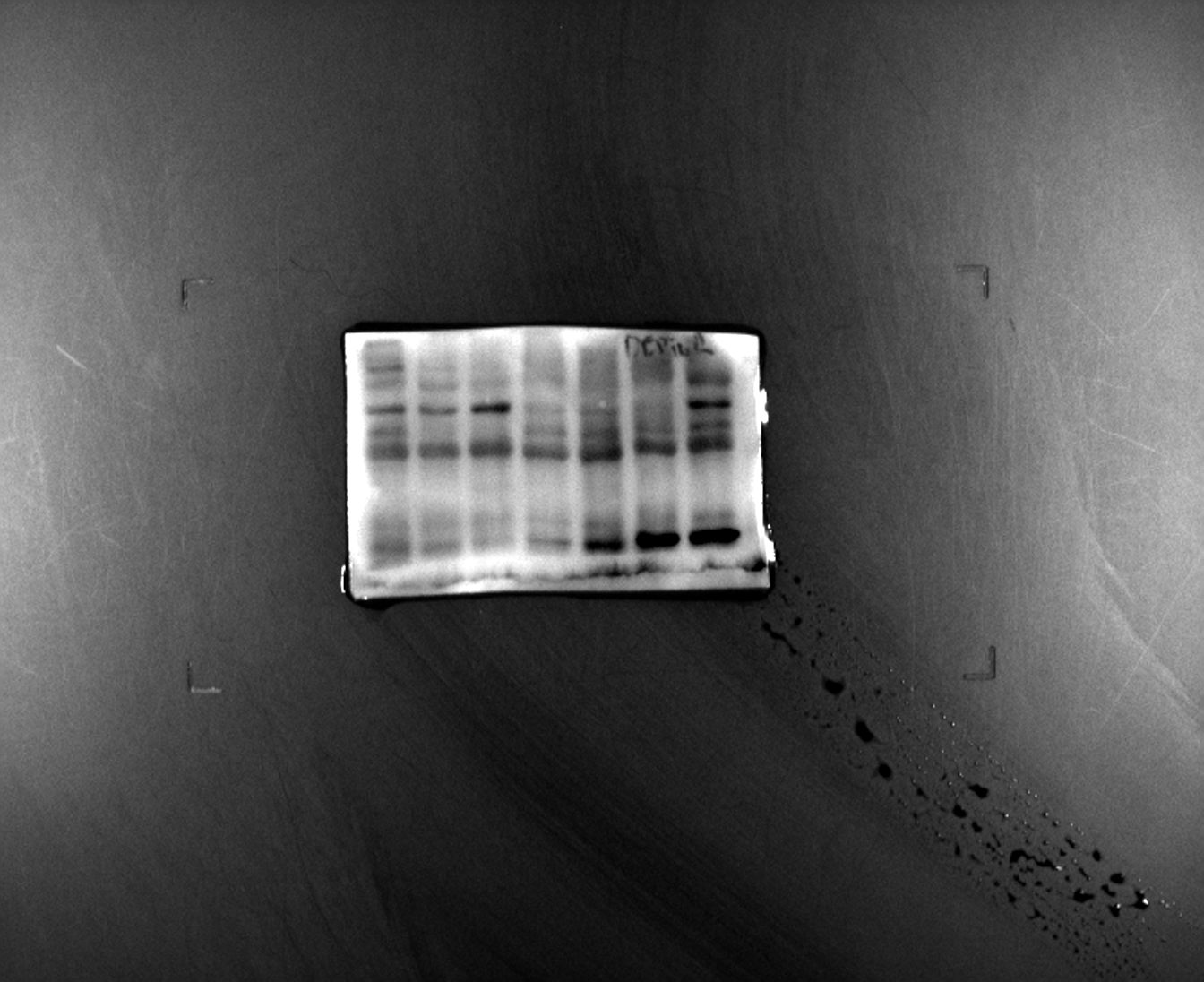


mTOR

p-mTOR

4EBP1

40

289

289

20

DEPTOR


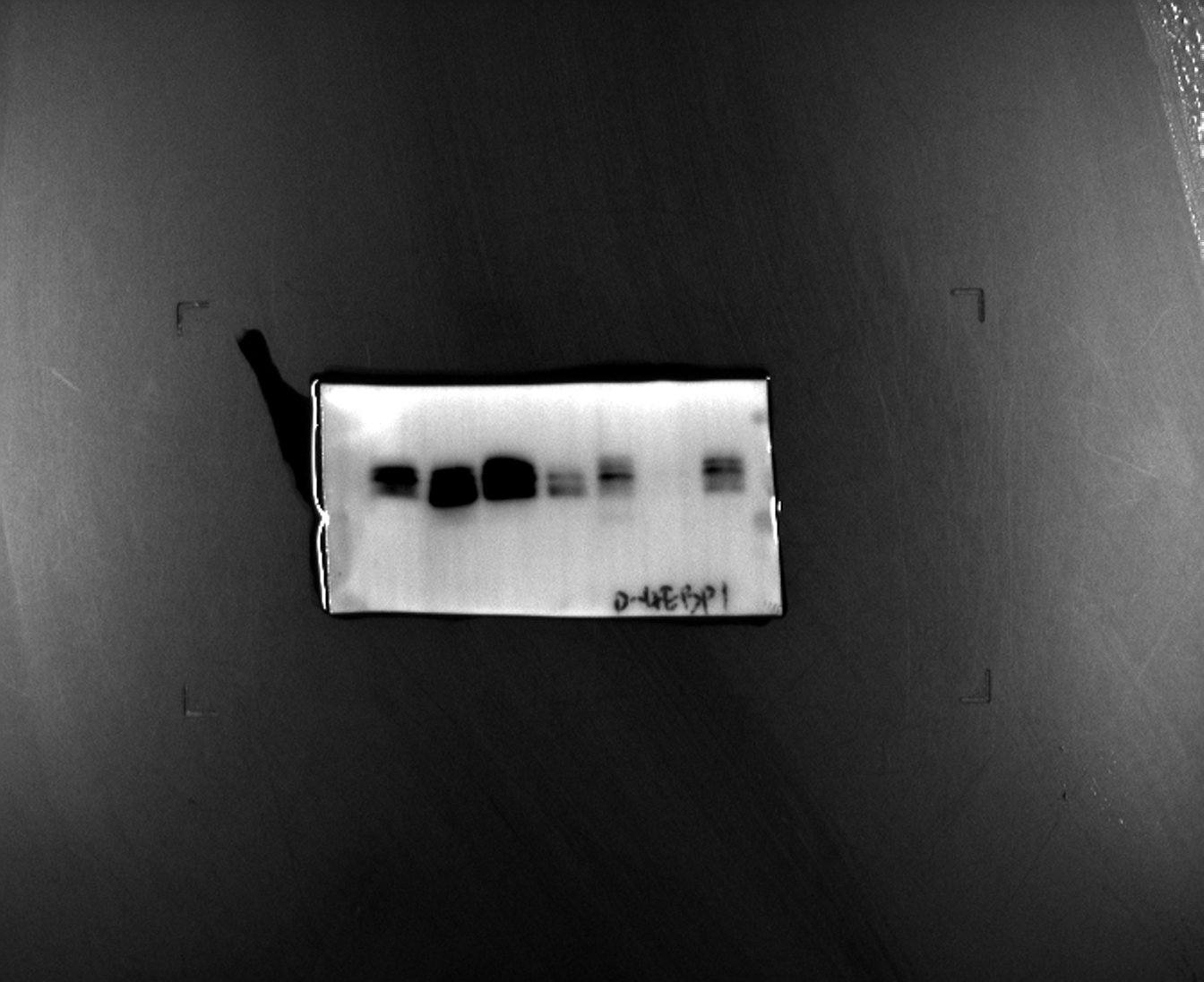


20

p-4EBP1


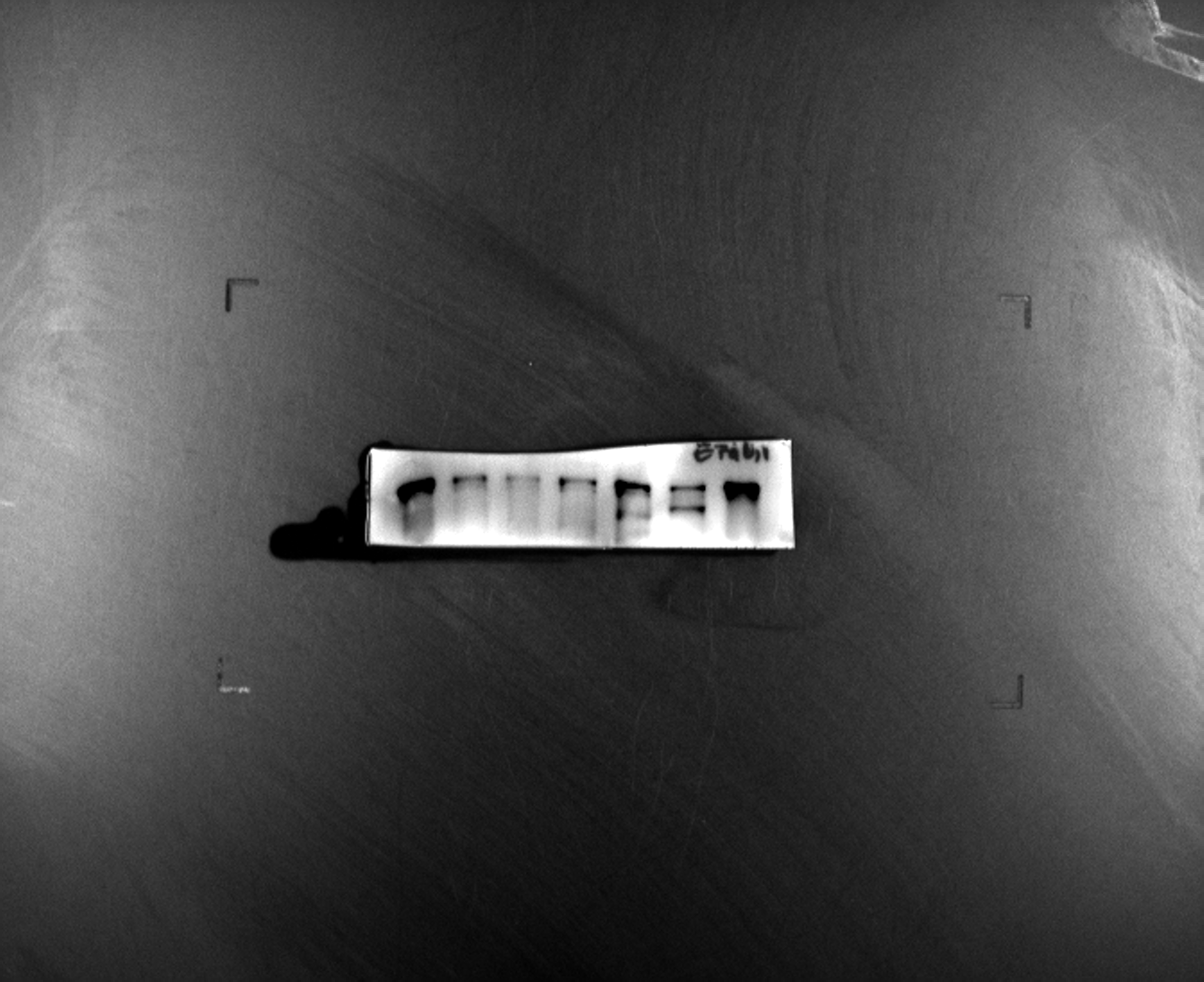

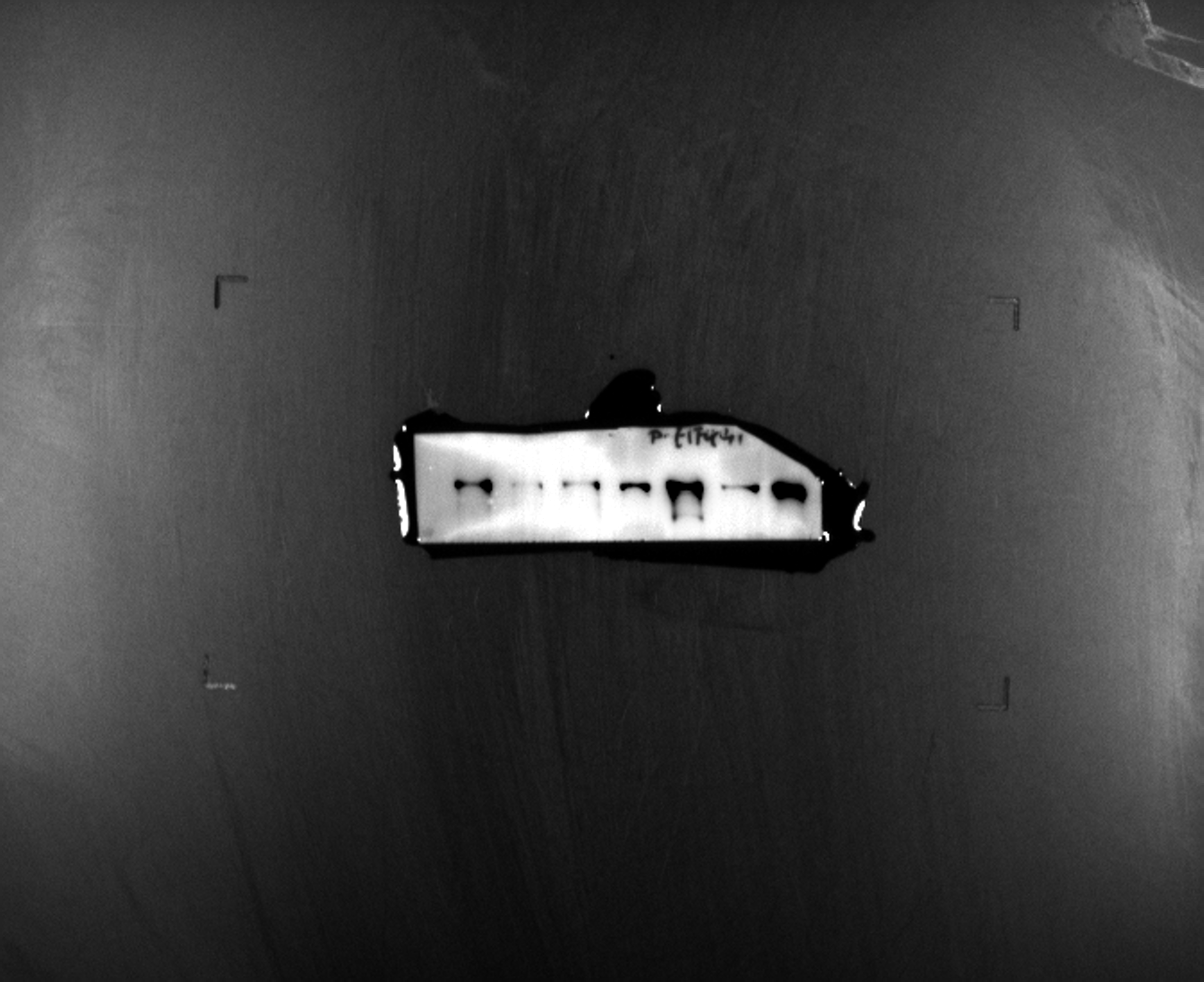

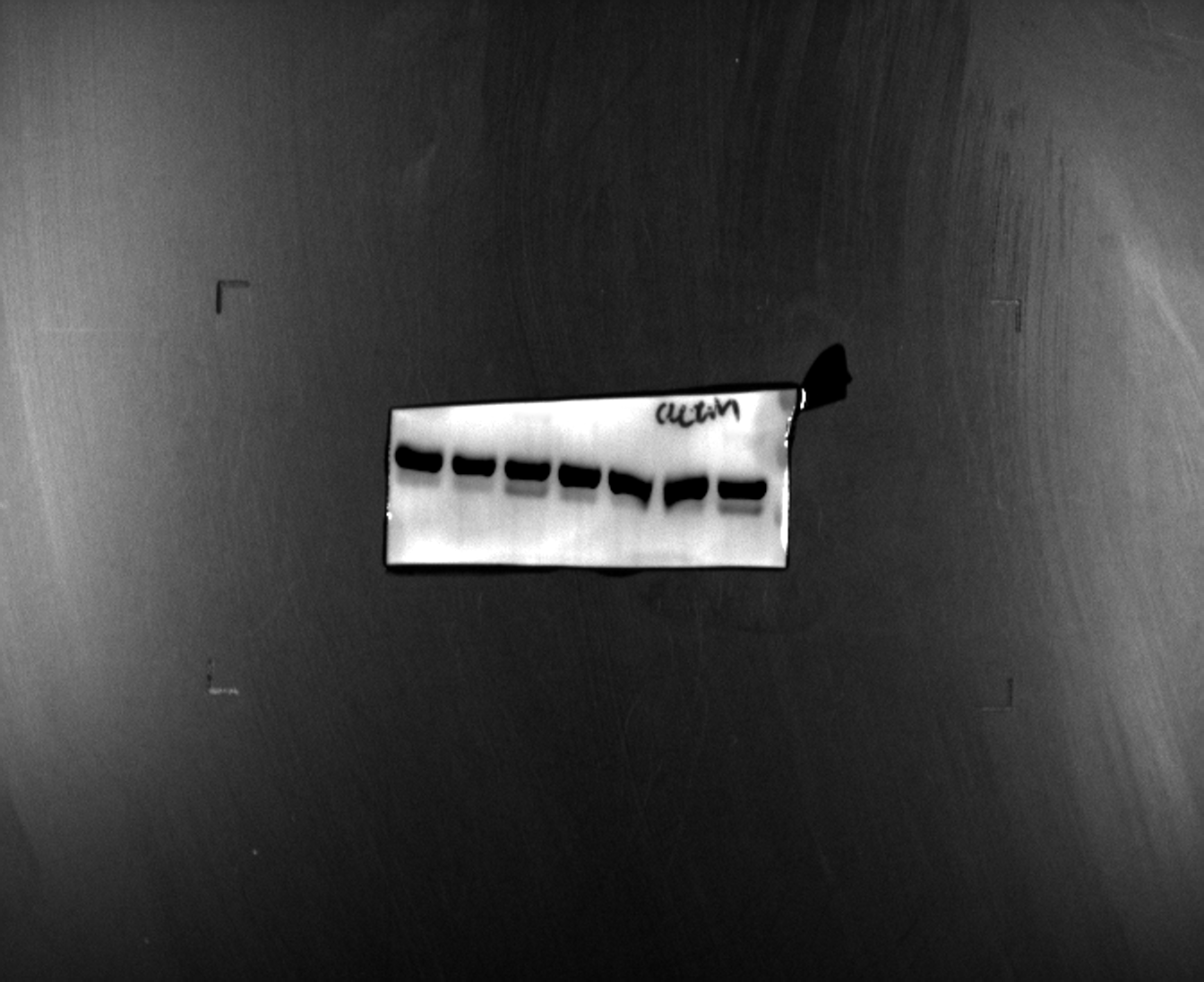


EIF4G1

220

p-EIF4G1

220

beta-actin

43

**Supplementary Tables**

**Table S1.** Clinical information of patients from Chen-cohort

**Table S2.** Clinical information of patients from CPTAC-cohort

**Table S3.** Clinical information of patients from TCGA-cohort

**Table S4.** Clinical information of patients from Xu-cohort

**Table S5.** PTM-SEA analysis in Chen-cohort

**Table S6.** Kinase-substrate enrichment analyses (KSEA) in Chen cohort

**Table S7.** Annotation of drugs screened by CMAP analysis
